# Synthesis of a novel resorcin[4]arene–glucose conjugate and its catalysis of the CuAAC reaction for the synthesis of 1,4-disubstituted 1,2,3-triazoles in water†

**DOI:** 10.1039/c9ra00972h

**Published:** 2019-03-29

**Authors:** Ali A. Husain, Kirpal S. Bisht

**Affiliations:** Department of Chemistry, University of South Florida 4202 East Fowler Avenue, CHE 205 Tampa FL 33620 USA kbisht@usf.edu

## Abstract

The Cu(i)-catalyzed azide–alkyne cycloaddition (CuAAC) in aqueous media using resorcin[4]arene glycoconjugate (RG) is reported. The eight β-d-glucopyranoside moieties constructed on the resorcin[4]arene upper rim provide a pseudo-saccharide cavity that offers a suitable host environment for water-insoluble hydrophobic azido and/or alkyne substrates in water. The utility of RG was established as an efficient inverse phase transfer catalyst for the CuAAC in water as a green approach for the synthesis of 1,4-disubstituted 1,2,3-triazole species. The catalytic utility of RG (1 mol%) was demonstrated in a multicomponent one-pot CuAAC for various azido/alkyne substrates. The RG acts as a molecular host and a micro-reactor resulting in the 1,4-disubstituted 1,2,3-triazoles in excellent yield.

## Abstract 2

The Cu(i)-catalyzed azide–alkyne cycloaddition (CuAAC) in aqueous media using resorcin[4]arene glycoconjugate (RG) is reported.

## Graphical abstract

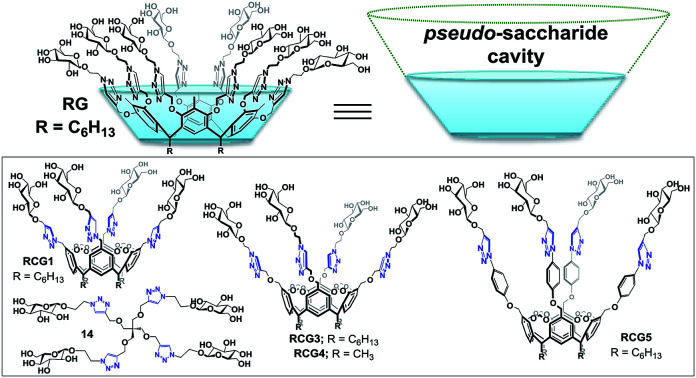

## Introduction

**Scheme 1:**
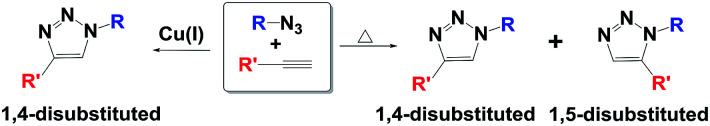
Azide–alkyne cycloaddition (AAC) reaction under different reaction conditions.

The classical Huisgen^1^ cycloaddition reaction for the synthesis of 1,2,3-triazole involves thermal 1,3-dipolar cycloaddition of organic azides with alkynes, though in low yield and mixed regioselectivity. Sharpless'^2^ and Meldal's^3^ research groups later independently developed the improved procedure involving the copper(i)-catalyzed Huisgen 1,3-dipolar cycloaddition reaction, which is the widely studied ‘click’ reaction (Scheme 1). The impact of the copper catalyzed azide–alkyne click reaction in various branches of science is increasing exponentially as evidenced from numerous recent reviews available in the literature since 2010.^4^

The three most common facile protocols for CuAAC include (i) use of copper(i) salts (mostly in organic solvents), (ii) the reduction of a copper(II to I), and (iii) oxidation of Cu(0 to I). Of the three protocols described above, the method employing *in situ* reduction of copper(ii) salts is known to be more practical and can be carried out in aqueous conditions. From review of literature, it is easy to conclude that water is an appropriate choice as a solvent for the CuSO_4_/sodium ascorbate catalyzed click protocol, which results in the formation of the triazole in high yields and with excellent regioselectivity. However, despite the efficiency of the CuAAC reaction, there are limitations to using the procedure especially when the substrates are not water-soluble. The protocol in essence requires deoxygenated conditions in the presence of mixed aprotic organic solvents such as THF, CH_3_CN, CH_2_Cl_2_, toluene, *etc.* and due to the oxidative tendency of the copper(i), a higher catalyst concentration throughout the reaction is needed. To stabilize the catalyst, several phosphine-based complexes and amine-based (bound with different heterocyclic donors) ligands have been used for rate acceleration.^5^ Additionally, a number of heterogeneous Cu catalysts,^6^ including amberlyst resin-supported,^7^ polymer-supported,^8^ and zeolite-supported^9^ have been explored to catalyze the triazole formation. To speed up the azide–alkyne reaction, use of surfactants and phase transfer catalyst,^10^ the microwave^11^ and ultrasound irradiations^12^ have also been reported.^13^

Recently, our research group reported resorcin[4]arene cavitand glycoconjugates (RCGs)^14^ as inverse phase transfer catalysts with abilities to catalyze organic reactions in aqueous media. We also reported on the RCGs ability to catalyze the formation of 1,4-disubstituted 1,2,3-triazoles in water without the addition of any co-organic solvents.^14^ It is noteworthy to mention that we were the first to establish the concept of the spatial directionality of β-d-glycopyranoside units on the resorcin[4]arene rigid structure “cavitand”. The RCGs possesses a unique molecular host system “pseudo-saccharide bucket” which can encapsulate organic substrates and catalyze chemical reactions in water.^14^

**Fig. 1:**
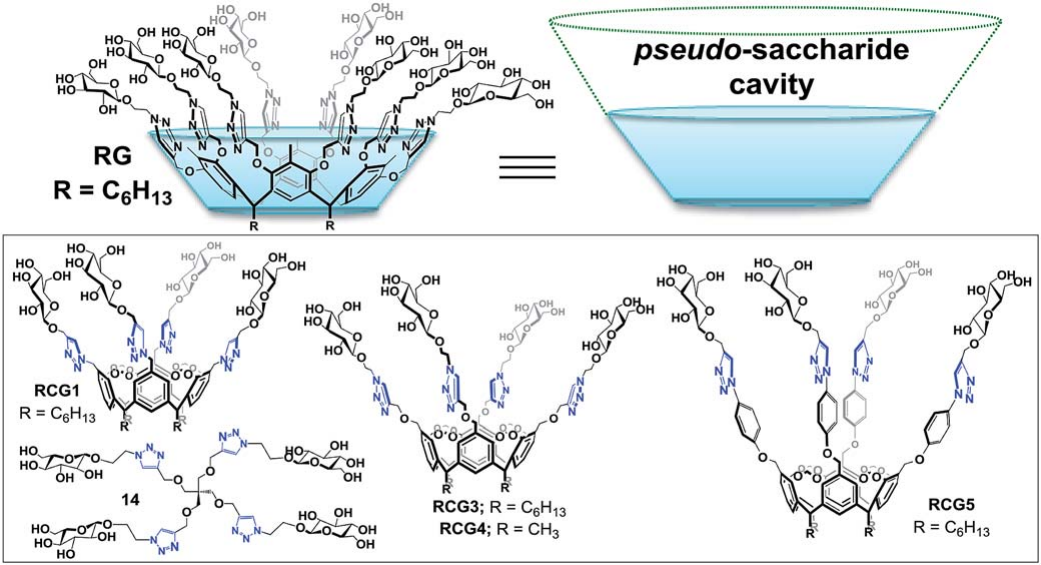
Resorcin[4]arene glycoconjugate (RG & RCGs^14^).

In this manuscript, we describe the synthesis of a resorcin[4]arene glycoconjugate (RG) (Fig. 1) and its application as a micro-reactor for the synthesis of 1,4-disubstituted 1,2,3-triazole species in aqueous media *via* the CuAAC reaction. RG structure consists of eight β-d-glucopyranoside moieties constructed on the phenolic parts on the resorcin[4]arene upper rim *via* multiple 1,4-disubstituted 1,2,3-triazole linkages. The eight arm resorcin[4]arene glycoconjugate offers an enlarged flexible pseudo-saccharide cavity that can act as a molecular vessel for water-insoluble azido and/or alkyne substrates in aqueous environment.

## Results and discussion

### Synthesis of eight arm resorcin[4]arene glycoconjugate (RG)

**Scheme 2:**
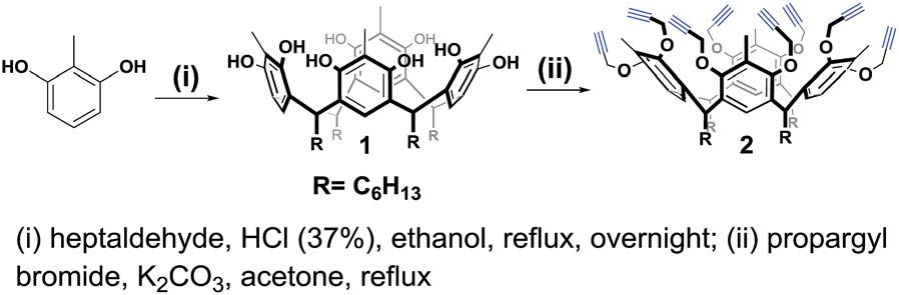
Synthesis of octa-alkyne resorcin[4]arene 2.

For the synthesis of the novel RG, resorcin[4]arene 1 (ref. 14 and 15) was first synthesized upon the acid-catalyzed cyclocondensation reaction of methyl resorcinol with heptanal. Compound 1 was then treated with propargyl bromide in the presence of potassium carbonate in refluxing acetone to achieve the octa-propargyl resorcin[4]arene intermediate 2 (Scheme 2).

**Fig. 2:**
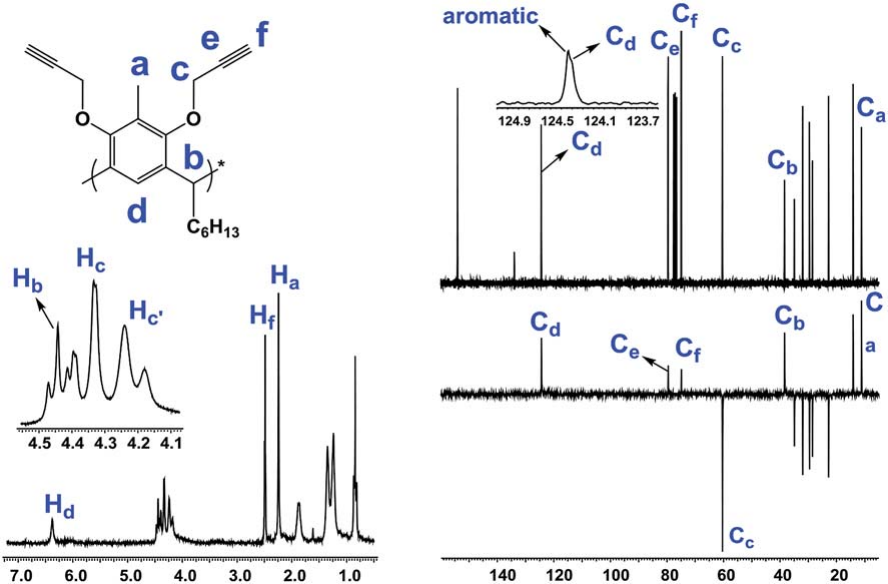
Partial ^1^H-, ^13^C- and DEPT NMR spectra (250 and 62.5 MHz, CDCl_3_) of 2.

Resorcin[4]arene 2 was characterized conclusively from its NMR and spectral data. In its ^1^H-NMR spectrum, the benzylic protons (–C*H*_3_, **H**_**a**_) were found as a singlet at 2.25 ppm. The alkyne protons (–C≡C*H*, **H**_**f**_) were observed as a triplet at 2.50 ppm (*J* = 2.4 Hz) and the propargyl methylene protons (–OC*H*_2_C≡, **H**_**c,c′**_) showed as two set of double doublets with *J* = 15.3 and 2.4 Hz at 4.20 and 4.36 ppm. Its ^13^C-NMR spectrum had resonances for the alkyne carbons **C**_**e**_ and **C**_**f**_ at 74.8 and 79.5 ppm, respectively, and the propargyl methylene carbons (–O*C*H_2_C≡, **C**_**c**_) were at 60.3 ppm (Fig. 2). The molecular formula (C_80_H_96_O_8_) of compound 2 was confirmed from its *m*/*z* measurement using ESI-Q-TOF HRMS: observed 1207.7034 (M + Na)^+^, calculated 1207.7003 (M + Na)^+^.

**Scheme 3:**
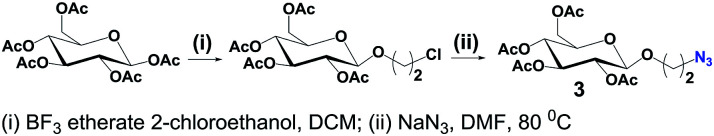
Synthesis of 3.

2-Azidoethyl β-d-glucopyranoside tetraacetate (3) was prepared following the typical procedures found in literature (Scheme 3) and unambiguously characterized.^14,16^

**Scheme 4:**
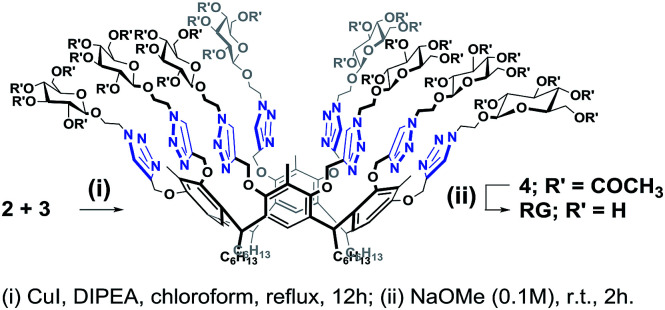
Synthesis of RG.

Octa-propargyl resorcin[4]arene 2 and azido glucopyranoside 3 were then coupled together *via* the CuAAC reaction. The reaction was carried out in refluxing chloroform in presence of CuI (10 mol%) and DIPEA (6.0 equiv.) to yield the octaacetoxy-RG (4) in excellent yield. Global deacetylation using NaOMe solution (0.1 M) resulted in RG in gram quantity (Scheme 4).

**Fig. 3:**
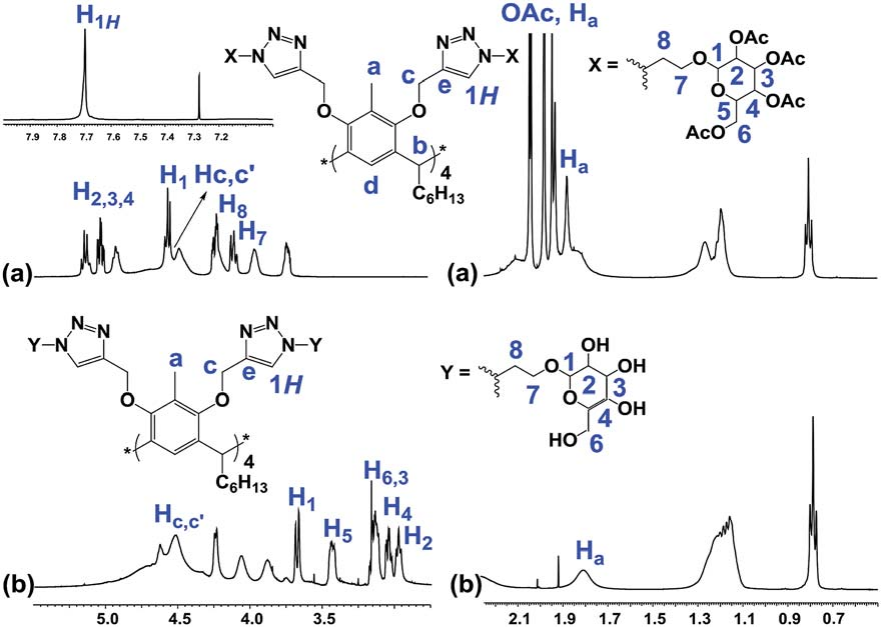
Partial ^1^H-NMR spectra of (a) 4 (500 MHz, CDCl_3_) and (b) RG (500 MHz, DMSO-d_6_).

The structures of the RG and its octaacetoxy precursor 4 were established from the respective ^1^H- and ^13^C-NMR data. Namely, the absence of the alkyne protons (**H**_**f**_), the emergence of the triazole protons (**1*H***, at 7.69 ppm) and the shift of the propargyl protons (**H**_**c**_, from 4.11 ppm to 4.50 ppm) confirmed the structure of 4 (Fig. 3). In the ^1^H-NMR spectra of RG (Fig. 3b), the disappearance of the acetate protons (–OCOC*H*_3_, **OAc**) from the region of 1.70–2.10 ppm was confirmatory of its structure. The molecular weight of the products {for 4*m*/*z* (ESI-Q-TOF): observed 2283.8955 (M + 2Na)^2+^, calculated 2283.8984 (M + 2Na)^2+^; for RG*m*/*z* (ESI-Q-TOF): observed 1611.7374 (M + 2Na)^2+^, calculated 1611.7294 (M + 2Na)^2+^} confirmed the respective molecular formula.

### Optimizing CuAAC in water catalyzed by RG

**Fig. 4:**
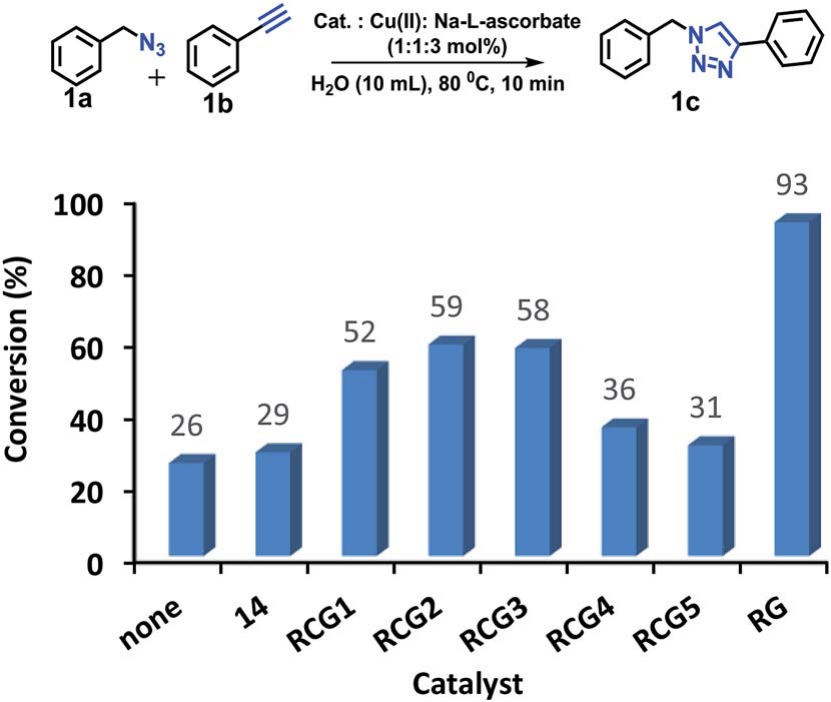
CuAAC of benzyl azide and phenyl acetylene at 80 °C. RCG catalyst structures in Fig. 1.

The CuAAC reactions was investigated to examine the catalytic activity in aqueous environment. For optimization, the CuAAC reaction of benzyl azide (1.0 mmol) with phenyl acetylene (1.05 equiv.) with/without the addition of a catalyst (1 mol%) was carried out (Fig. 4). The reactions were performed in the presence of copper sulfate (1 mol%) and sodium l-ascorbate (3 mol%) in 10 mL of distilled deionized water at 80 °C (Fig. 4). The chemical structures and synthetic procedure for RCG catalysts evaluated in Fig. 4 have been previously reported by our research group.^14^

The coupling reaction was much slower in absence of added RCG catalyst and in presence of compound 14 (Fig. 1),^14^ which lacks the spatial directionality of the RCGs. While the RCGs catalyzed the CuAAC reaction between benzyl azide and phenyl acetylene, there were significant differences in substrate conversion to suggest a dependence of catalytic activity on chemical structure (Fig. 4). Remarkably, the CuAAC reaction was almost completed with more than 93% conversion only in 10 minutes when RG (1 mol%) was added but only 26% conversion was observed in its absence (no catalyst). Obviously, the fast CuAAC in the presence of RG indicates that it provides a unique molecular environment that is capable of catalyzing the CuAAC reaction efficiently.

### Scoping the CuAAC in water using RG

To scope the CuAAC reactions catalyzed by RG in aqueous media, we have investigated coupling of substituted benzyl azides 1a–4a with aromatic and aliphatic alkynes 1b–4b (Table 1).

**Table 1:** CuAAC of substituted benzyl azides 1a–4a and alkyne substrates 1b–4b in the presence of RG^a^

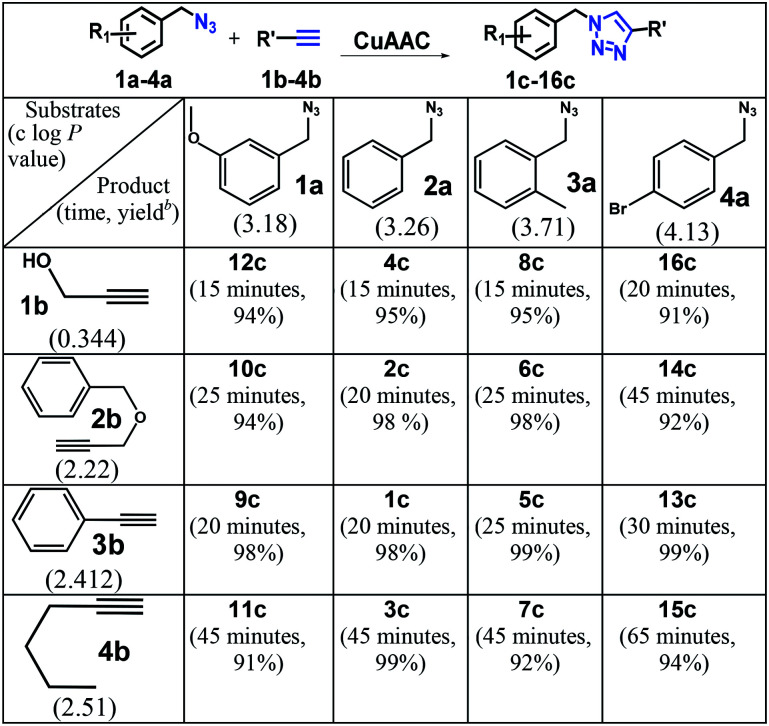

a Reaction condition: benzyl azide derivative (1 mmol) and alkyne substrates (1.05 mmol), Cu(ii) (1 mol%), Na-l-ascorbate (3 mol%), RG (1 mol%), water (10 mL), 80 °C.

b Isolated yield.

As recorded in Table 1, the RG catalyzed CuAAC reactions of a variety of substituted benzyl azides in water and all reactions led to the desired 1,4-disubstituted 1,2,3-triazole products (2c–16c) in high yields (>90% isolated). Not surprisingly, it was determined that the reaction took longer to reach completion as the hydrophobicity of the substrate pair increased, as evidenced by their clog *P* values (Table 1). For example, the coupling between the 4-bromo benzyl azide (4a, clog *P* = 4.13) and 1-hexyne (4b, clog *P* = 2.51) took nearly 65 minutes to completion while the reactions between propargyl alcohol (1b, clog *P* = −0.34) and 3-methoxy benzyl azide (1a, clog *P* = 3.18) was completed in 15 minutes.

To further evaluate the effectiveness of the RG as a catalyst in CuAAC of hydrophobic substrates, we carried out the coupling of phenyl acetylene (3b) with alkylated *ortho*-azido phenols (5a–9a) of increasing steric bulk and clog *P* values from 2.55–4.95. A comparative study of the CuAAC reaction in absence and presence of the RG is shown in Table 2.

**Table 2:** CuAAC of *o*-azido phenol derivatives (5a–9a) with phenyl acetylene 1b with/without RG^a^

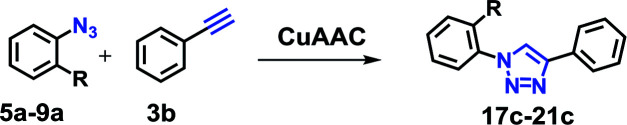					
Entry	Azide (clog *P* value)	Product	Time (min)	Catalyst (% yield)^b^	
				None	RG
1	5a R = OH (2.55)	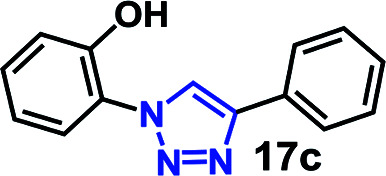	30	64	96
2	6a R = OMe (3.18)	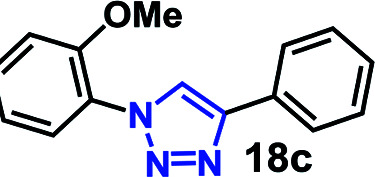	30	22	97
3	7a R = OEt (3.71)	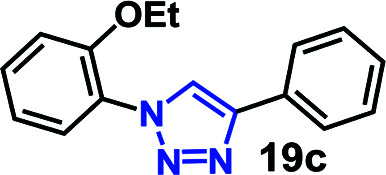	30	16	98
4	8a R = OBu (4.77)	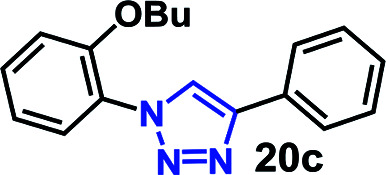	30	12	98
5	9a R = OBn (4.95)	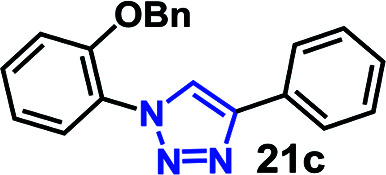	30	<10	99

a Reaction condition: azidophenol derivative (1 mmol) and phenyl acetylene 1b (1.05 mmol), Cu(ii) (1 mol%), Na-l-ascorbate (3 mol%), cat. (1 mol%), water (10 mL), 80 °C.

b Isolated yield.

As expected, the coupling of *o*-azido phenols (5a, clog *P* = 2.44) with phenyl acetylene 1b catalyzed by RG resulted in triazole 17c in 96% yield and even without added catalyst the yield was 64% due to its higher hydrophilicity (entry 1). However, replacing the hydroxyl functionality with alkoxy groups, *i.e.* –OMe, –OEt, –OBu, –OPh of progressively higher bulk, hydrophobicity, and clog *P* value led to much slower reactions in absence of the RG. The reactions catalyzed by the RG resulted in near quantitative conversion in about 30 minutes irrespective of the bulk or hydrophobicity of the azide substrates, attesting to its effectiveness as a catalyst in the CuAAC reactions in water even for the much bulkier and hydrophobic substrates.

### Di-CuAAC reactions in water using RG

Simultaneous multiple CuAAC reactions have found interest in synthesis of the polymers and dendrimers and the need for efficient catalyst that can catalyze reactions in water has never been greater.^17^ We have therefore investigated RG for catalyzing the di-CuAAC reactions in water of di-propargyl benzene derivatives 5b–7b with substituted benzyl azides 1a–4a (Table 3). Interestingly, the di-CuAAC reaction were completed within 45 min resulting in the desired bis-1,2,3-triazole products 22c–33c in gram quantities.

**Table 3:** Di-CuAAC of di-alkynes 5b–7b with benzyl azide derivatives 1a–4a in water in the presence of RG^a^^,^^b^

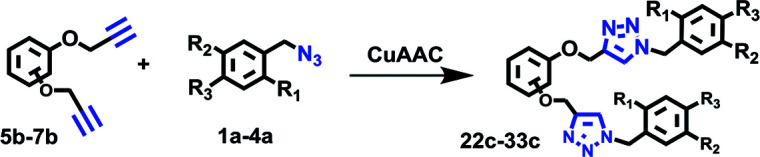
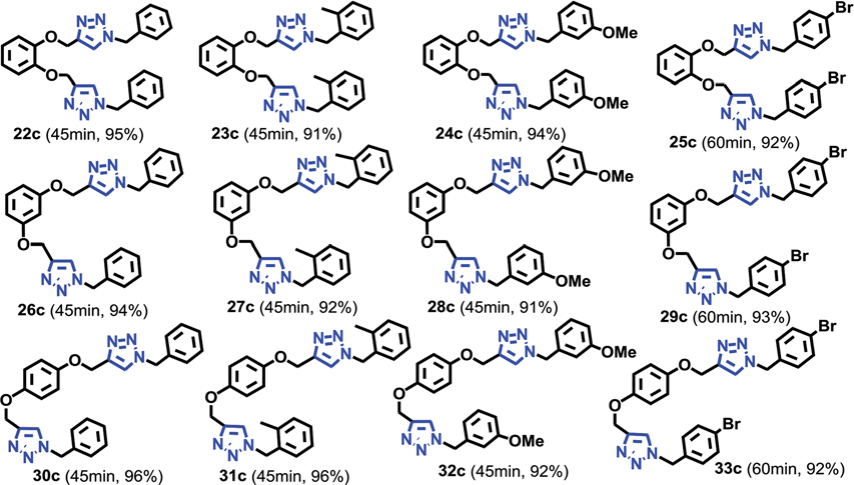

a Reaction condition: di-alkyne derivative (1 mmol) and azides (2.1 mmol), Cu(ii) (1 mol%), Na-l-ascorbate (3 mol%), RG (1 mol%), water (10 mL), 45 min, 80 °C.

b Isolated yields.

### Multicomponent one-pot CuAAC reactions in water

Organic azides are not always considered safe for handling because of their toxic and shock sensitive nature and there have been alternative methods employed for their *in situ* synthesis. The azides can be prepared from their corresponding halides upon the addition of sodium azide. Hence, a multicomponent one-pot CuAAC reaction between *in situ* generated azide from its corresponding precursor and alkyne is highly desirable.^18^

We have investigated the multicomponent one-pot CuAAC reactions of phenyl acetylene 3b with the aryl bromides and sodium azide catalyzed by the RG. Complete conversion were accomplished within 25–55 minutes even for a bulkier aryl system (naphthyl) to achieve the 1,2,3-triazoles in excellent isolated yield (90–96%) (Table 4, entries 1–5). In addition, reaction with α-bromo acetophenones (entries 6 and 7), α-bromoesters (entry 8) and ally bromide (entry 9) led to quantitative conversion within 25–30 min to the desired triazoles 35c–37c in 91–95% isolated yield. However, the multi component CuAAC failed when saturated aliphatic bromides were used as substrates, *i.e*., butyl, heptyl and dodecyl bromides (entries 10–12). We suspect the π–π interactions play an important role in the encapsulation, in water, of the substrates molecule in the hydrophobic cavity of the RG and the lack of these interactions in the aliphatic alkyl bromide does not allow their encapsulation in the RG cavity and hence no observed reaction.

**Table 4:** Multicomponent one-pot CuAAC of phenyl acetylene, aryl/alkyl bromides and sodium azides in the presence of RG^a^

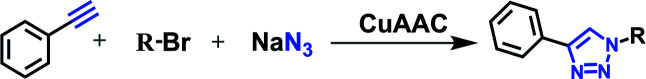			
Entry	Product	Time (min)	Yield^b^ (%)
1	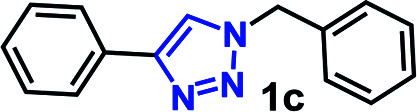	25	92
2	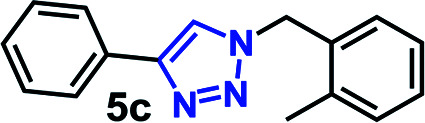	30	94
3	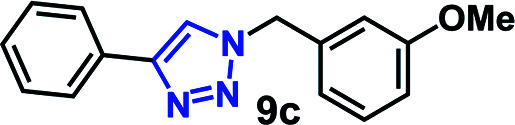	30	96
4	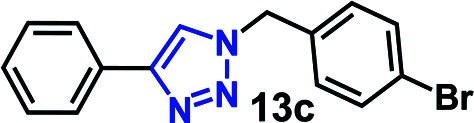	50	90
5	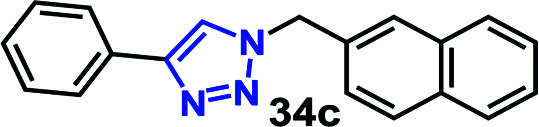	55	91
6	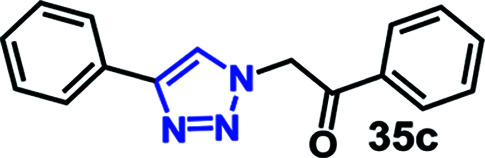	30	92
7	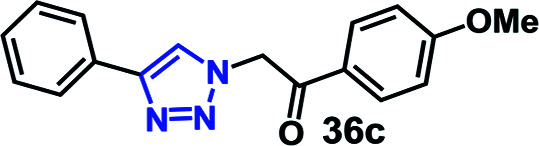	30	95
8	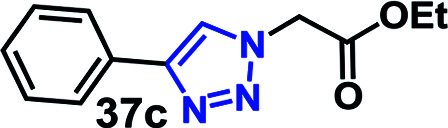	25	93
9	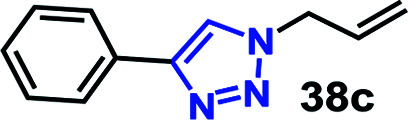	25	91
10	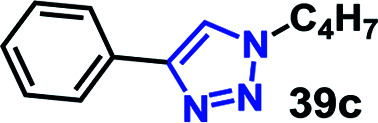	200	<10
11	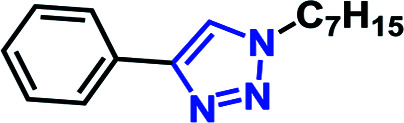	200	N/A
12	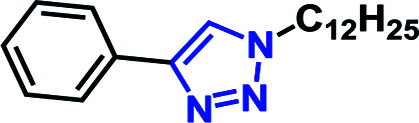	200	N/A

a Reaction condition: phenyl acetylene (1 mmol), bromide derivative (1.05 mmol) and sodium azide (1.1 mmol), RG (1 mol%), water (10 mL), 80 °C.

b Isolated yields, N/A = not isolated.

### Inclusion complex of RG with benzyl azide (2a) and phenyl acetylene (3b) in D_2_O

**Fig. 5:**
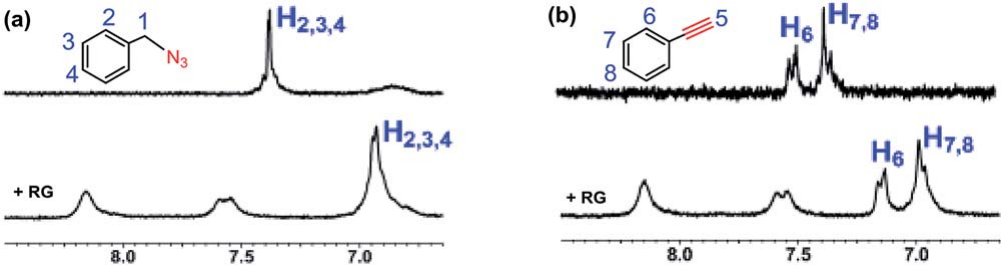
Partial ^1^H-NMR spectra (250 MHz, D_2_O, 2 mM) at 25 °C of (a) benzyl azide and benzyl azide + RG (1 : 1); (b) phenylacetylene and phenylacetylene + RG (1 : 1).

We set out to probe the encapsulation of the substrates by the RG. Specifically, we have studied the encapsulation of benzyl azide and phenyl acetylene by RG using NMR. The ^1^H-NMR spectra of the guest (2a & 3b) were recorded in the presence of the host (RG) in a 1 : 1 molar ratio for 2 mM concentrated solution in D_2_O at 25 °C (Fig. 5). In the ^1^H-NMR, the aromatic protons (H_2_, H_3_, H_4_) in 2a were shifted up field from 7.35 ppm to 6.90 ppm upon addition of the RG (Fig. 5a). Similarly, the aromatic protons (H_6_, H_7_, H_8_) in 3b were shifted upfield from 7.35 and 7.45 ppm to 6.98 and 7.15 ppm, respectively upon addition of RG (Fig. 5b). The shielding of the guest ^1^H NMR resonances upon addition of RG indicated their encapsulation in the RG cavity. The encapsulation of the substrates in the RG cavity may explain the catalytic activity of the RG. In addition, the observation that the CuAAC reactions of the aromatic and π bond containing azides were accelerated in the presence of RG (Table 4), the encapsulation of the guest most likely involves π–π host–guest interactions.

### Proposed mechanism for the CuAAC using RG

**Fig. 6:**
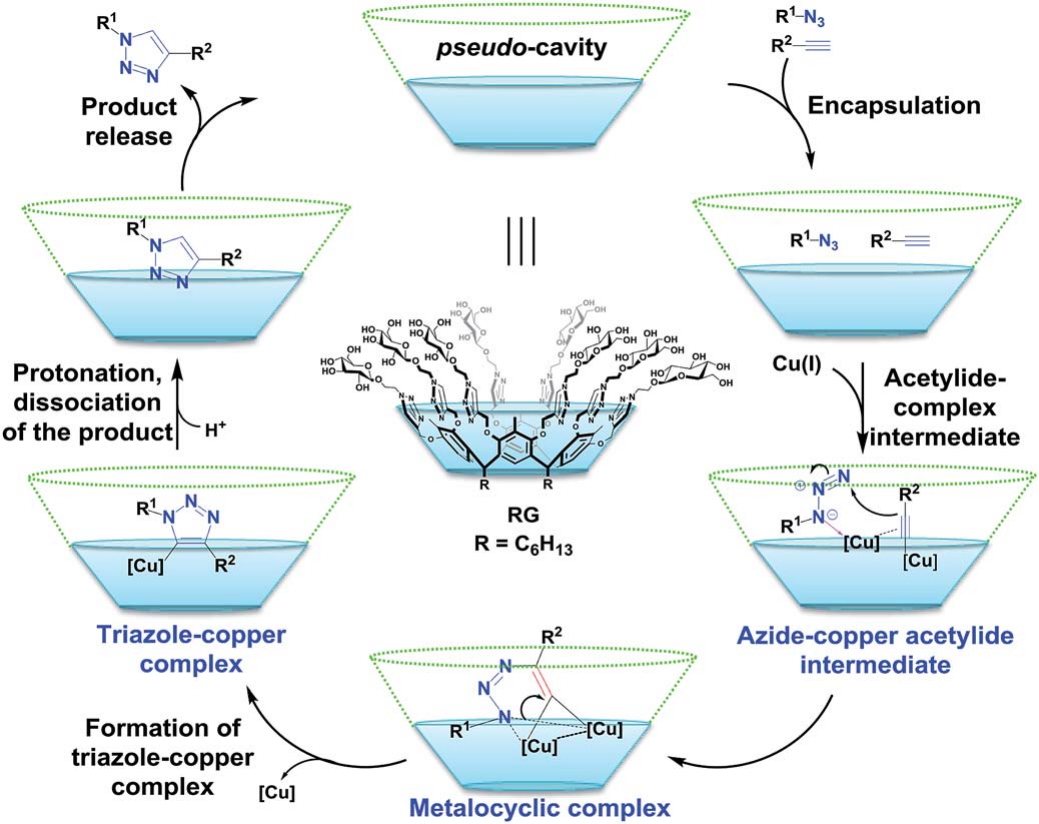
Proposed mechanism for the CuAAC in aqueous media catalyzed by RG.

Based upon the reaction catalysed and the encapsulation observed, the proposed mechanism for the CuAAC reaction catalyzed by RG may proceed *via* step shown in Fig. 6. Starting with inclusion of the alkyne and azide substrates in the pseudo-β-d-glucopyranoside cavity of RG. The closed proximity of the alkyne and azide in the presence of Cu(i) catalyst accelerate the cycloaddition process resulting in the copper–triazole complex followed by protonation and dissociation of the desired 1,4-di substituted 1,2,3-triazole (Fig. 6).^19^ The binding of the Cu(i) to the multiple triazoles in the RG may also facilitate the coupling reaction inside the RG cavity.

## Conclusion

In conclusion, a new resorcinarene sugar conjugate (RG) is reported. It was determined that RG is an efficient catalyst for the CuAAC reaction in water at only 1 mol%. Based upon the reaction catalysis and the encapsulation observed, RG catalytic mechanism, we believe, begins with inclusion of the alkyne and azide substrates resulting in the copper–triazole complex followed by protonation and dissociation to the desired 1,4-di substituted 1,2,3-triazole. RG was found to catalyze the coupling of the alkyne/azide pair and also of the multicomponent alkyl bromide/sodium azide/alkyne to the 1,4-disubstituted 1,2,3-triazole products in excellent yield within short period of time in water.

## Experimental section

### General

^1^H- and ^13^C-NMR spectra were recorded on a Bruker DRX-250, a Inova-400 and a DD-500 spectrometers. Sample concentrations were about 10% (w/v) in CDCl_3_ or DMSO-d_6_ and the *J* values are given in Hz. The mass spectral analyses were performed on an Aligent Technologies 6540 UHD Accurate-Mass Q-TOF LC/MS. The clog *P* values were calculated using ChemDraw Professional, Version 15.1.0.144.

### Materials and reagents

All reagents were used with no further purification unless otherwise specified. 2-Methyl resorcinol (98%) was purchased from Acros Organics Chemical Company. Octa-hydroxy resorcin[4]arene (1) and 2-azidoethyl β-d-glucopyranoside tetraacetate (3) were synthesized following synthetic procedures reported previously.^14–16^

#### Octa-propargyl resorcin[4]arene (2)

Compound 1 (5 g, 5.7 mmol) was dissolved in 110 mL acetone. Potassium carbonate (6.26 g, 45 mmol) was added into the solution and allowed to stir for 10 min at room temperature. Propargyl bromide (8.1 mL, 90 mmol) was then added and the reaction mixture was refluxed overnight. After completion, the reaction mixture was cooled at room temperature and the salt was filtered out followed by the concentration of acetone. The product was purified by column chromatography as a pale yellow solid using 10% EA/hexane yielding 6.04 g (90%) yield. ^1^H NMR (250 MHz, CDCl_3_) *δ* = 0.85 (t, *J* = 6.5 Hz, 12H), 1.25–1.36 (m, 16H), 1.36 (m, 16H), 1.82–1.94 (m, 8H), 2.25 (s, 12H), 2.50 (t, *J* = 2.4 Hz, 4H), 4.11 (d, *J* = 15.3 Hz, 8H), 4.25 (d, *J* = 15.3 Hz, 8H), 4.45 (t, *J* = 7.1 Hz, 4H), 6.39 (s, 4H); ^13^C NMR (62.5 MHz, CDCl_3_) *δ* = 11.1, 14.0, 22.7, 28.5, 29.5, 31.9, 34.9, 38.3, 60.3, 74.8, 79.5, 124.3, 124.3, 133.9, 154.0; HRMS [M + Na]^+^ calcd for C_80_H_96_O_8_Na 1207.7003, found 1207.7034.

#### Octa-sugar acetate resorcin[4]arene (6)

Compound 2 (1 g, 0.84 mmol) was dissolved in 30 mL chloroform. CuI (32 mg, 0.17 mmol) and DIPEA (0.88 mL, 5 mmol) were then added to the solution. Sugar azide 3 (5.6 g, 13.5 mmol) was then added to the solution. The reaction mixture was then refluxed overnight. After completion, the reaction was worked up using ammonium hydroxide solution and the organic phase was extracted and dried using Na_2_SO_4_. Chloroform was evaporated and the product was purified by column chromatography using 5% MeOH/DCM in order to separate the product from the excess sugar azide. The product was collected as yellow oil that solidified slowly to pale yellow solid in 3.48 g (91%) yield. ^1^H NMR (500 MHz, CDCl_3_) *δ* = 0.80 (t, *J* = 6.6 Hz, 12H), 1.15–1.35 (m, 32H), 1.78–1.85 (bs, 12H), 1.88 (s, 12H), 1.93 (s, 12H), 1.98 (s, 24H), 2.04 (s, 12H), 2.04 (s, 12H), 2.07–2.16 (bs, 12H), 2.37 (bm, 8H), 3.71–3.77 (m, 8H), 3.91–4.00 (m, 8H), 4.06–4.14 (m, 8H), 4.22 (t, *J* = 2.9 Hz, 8H), 4.25 (dd, *J* = 2.9, 4.4 Hz, 4H), 4.49 (bs, 16H), 4.55 (d, *J* = 8.1 Hz, 8H), 4.58 (t, *J* = 8.1 Hz, 4H), 4.93 (t, *J* = 7.7 Hz, 8H), 5.03 (ddd, *J* = 3.3, 9.5, 13.2 Hz, 8H), 5.13 (q, *J* = 9.9, 19.1 Hz, 8H), 7.69 (s, 8H); ^13^C NMR (125 MHz, CDCl_3_) *δ* = 10.7, 14.0, 20.3, 20.4, 20.4, 20.6, 22.7, 28.6, 29.6, 31.8, 35.2, 38.0, 49.5, 61.6, 65.9, 67.5, 68.1, 70.7, 71.7, 72.4, 100.4, 123.7, 124.1, 124.2, 144.3, 144.5, 169.3, 170.0, 170.5; HRMS [M + 2Na]^2+^ calcd for C_208_H_280_N_24_O_88_Na_2_ 2283.8984, found 2283.8955.

#### Resorcin[4]arene glycoconjugate (RG)

Acetylated sugar resorcin[4]arene 6 (1 g) was dissolved in (0.1 M) sodium methoxide solution. The reaction mixture was allowed to stir at room temperature for 4 h. After completion, the reaction mixture was neutralized using Dowex 50W-X8. This was followed by filtration and concentration of methanol with no further purification to afford RG as a pale yellow solid in 92%; ^1^H NMR (250 MHz, DMSO-d_6_) *δ* = 0.79 (t, *J* = 6.6 Hz, 12H), 1.14–1.36 (m, 32H), 2.27 (bm, 8H), 2.27 (bs, 12H), 2.98 (t, *J* = 8.1 Hz, 8H), 3.05 (ddd, *J* = 3.3, 9.2, 18.3 Hz, 8H), 3.11–3.19 (m, 16H), 3.41–3.47 (m, 8H), 3.67 (d, *J* = 10.6 Hz, 8H), 3.89 (bs, 8H), 4.07 (bs, 8H), 4.24 (d, *J* = 7.0 Hz, 8H), 4.53 (bs, 24H), 4.63 (bs, 16H), 7.93–8.39 (bs, 12H); ^13^C NMR (62.5 MHz, DMSO-d_6_) *δ* = 11.1, 14.0, 22.2, 28.1, 29.1, 31.4, 34.8, 37.8, 49.6, 61.1, 65.4, 67.3, 70.0, 73.3, 76.6, 77.0, 102.9, 123.8, 124.3, 124.7, 142.9, 143.1; HRMS [M + 2Na]^2+^ calcd for C_144_H_216_N_24_O_56_Na_2_ 1611.7294, found 1611.7374.

### Typical procedure for screening and scoping the CuAAC in water catalyzed by RG

Aryl azide (1 mmol) and aryl/alkyl alkyne (1.05 equiv.) were added to a solution of copper(ii) sulfate pentahydrates, Na-ascorbate and RG (1 : 3 : 1 mol%) in 10 mL de-ionized distilled water. The reaction mixture was then heated to 80 °C for 15–65 min. After completion, the solid reaction product was filtered off, dried, and weighed to calculate the isolated yield. When the product was not solid (10c and 11c), the reaction mixture was extracted with DCM (2 × 5 mL). The combined organic layer was collected and dried over MgSO_4_. DCM was then removed using a rotary evaporator and ^1^H-NMR was taken in CDCl_3_ or DMSO-d_6_.

### Typical procedure for three component one-pot CuAAC catalyzed by RG

Phenyl acetylene 1b (1 mmol) and aryl/alkyl bromides (1.05 equiv.) with sodium azide (1.1 equiv) were added to a solution of copper(ii) sulfate pentahydrates, Na-ascorbate and RG (1 : 3 : 1 mol%) in 10 mL de-ionized distilled water. The reaction mixture was then heated to 80 °C for 20–55 min. After completion, the solid reaction product was filtered off, dried, and weighed to calculate the isolated yield. When the conversion was low (entries 10–12, Table 4), the reaction mixture was extracted with DCM (2 × 5 mL). The combined organic layer was collected and dried over MgSO_4_. The solvent was then removed using a rotary evaporator and ^1^H-NMR was taken in CDCl_3_.

#### 4-((Benzyloxy)methyl)-1-(2-methylbenzyl)-1*H*-1,2,3-triazole (6c)

White solid (98%); ^1^H NMR (400 MHz, CDCl_3_) *δ* 2.25 (s, 3H), 4.55 (s, 2H), 4.62 (s, 2H), 5.48 (s, 2H), 7.11–7.13 (m, 1H), 7.15–7.21 (m, 2H), 7.24 (d, *J* = 7.0 Hz, 2H), 7.29 (m, 4H), 7.33 (s, 1H); ^13^C NMR (100 MHz, CDCl_3_) *δ* 18.9, 52.2, 63.6, 72.4, 126.5, 127.7, 127.8, 128.3, 129.0, 129.4, 130.9, 132.3, 136.8, 137.6.

#### 4-Butyl-1-(2-methylbenzyl)-1*H*-1,2,3-triazole (7c)

White solid (90%); ^1^H NMR (400 MHz, CDCl_3_) *δ* 0.87 (t, *J* = 7 Hz, 3H), 1.27–1.36 (m, 2H), 1.54–1.61 (m, 2H), 2.23 (s, 3H), 2.64 (t, *J* = 7.8 Hz, 2H), 5.45 (s, 2H), 7.15–7.18 (m, 2H) 7.22–7.26 (m, 1H); ^13^C NMR (100 MHz, CDCl_3_) *δ* 13.7, 18.9, 22.2, 25.3, 31.4, 51.0, 120.2, 126.5, 128.8, 129.1, 130.8, 132.7, 136.8.

#### 4-((Benzyloxy)methyl)-1-(3-methoxybenzyl)-1*H*-1,2,3-triazole (10c)

Brown oil (94%); ^1^H NMR (400 MHz, CDCl_3_) *δ* 3.71 (s, 2H), 4.54 (s, 2H), 4.62 (s, 2H), 5.40 (s, 2H), 6.77 (s, 1H), 6.79–6.85 (m, 2H), 7.20–7.26 (m, 2H), 7.28–7.30 (m, 3H), 7.47 (s, 1H); ^13^C NMR (100 MHz, CDCl_3_) *δ* 53.6, 54.9, 63.3, 72.1, 113.4, 113.7, 119.9, 122.3, 127.4, 127.5, 128.0, 135.8, 137.5, 145.1, 159.7.

#### 4-Butyl-1-(3-methoxybenzyl)-1*H*-1,2,3-triazole (11c)

Brown oil (91%) yield; ^1^H NMR (400 MHz, CDCl_3_) *δ* 0.78 (t, *J* = 7.3 Hz, 3H), 1.19–1.28 (m, 2H), 1.46–1.54 (m, 2H), 2.56 (t, *J* = 7.7 Hz, 2H), 3.61 (s, 3H), 5.31 (s, 2H), 6.66 (s, 1H), 6.71 (t, *J* = 9.0 Hz, 2H), 7.12 (t, *J* = 7.9 Hz, 1H), 7.19 (s, 1H); ^13^C NMR (100 MHz, CDCl_3_) *δ* 13.3, 21.8, 24.9, 31.0, 53.2, 54.7, 113.0, 113.4, 119.6, 120.3, 129.5, 136.2, 148.2, 159.5.

#### 1-(2-Ethoxyphenyl)-4-phenyl-1*H*-1,2,3-triazole (19c)

White solid (98%); ^1^H NMR (400 MHz, CDCl_3_) *δ* 1.37 (t, *J* = 6.9 Hz, 3H), 4.08 (q, *J* = 6.9 Hz, 2H), 7.04 (m, 2H), 7.34 (m, 2H), 7.43 (t, *J* = 7.5 Hz, 2H), 7.81 (d, *J* = 7.8 Hz, 1H), 7.89 (d, *J* = 7.6 Hz, 2H), 8.38 (s, 1H); ^13^C NMR (100 MHz, CDCl_3_) *δ* 14.5, 64.6, 113.2, 121.0, 121.6, 125.0, 125.6, 126.3, 127.9, 128.7, 129.8, 130.7, 147.0, 150.1.

#### 1-(2-Butoxyphenyl)-4-phenyl-1*H*-1,2,3-triazole (20c)

White solid (98%); ^1^H NMR (400 MHz, CDCl_3_) *δ* 0.93 (t, *J* = 6.8 Hz, 3H), 1.40–1.47 (m, 2H), 1.72–1.78 (m, 2H), 4.05 (t, *J* = 6.3 Hz, 2H), 7.08 (t, *J* = 8.9 Hz, 2H), 7.32–7.39 (m, 2H), 7.44 (t, *J* = 8.0 Hz, 2H), 7.83 (d, *J* = 7.8 Hz, 1H) 7.88 (d, *J* = 8.1 Hz, 2H), 8.36 (s, 1H); ^13^C NMR (100 MHz, CDCl_3_) *δ* 13.7, 19.2, 31.0, 68.7, 113.2, 121.0, 121.7, 125.2, 125.7, 126.4, 128.0, 128.8, 129.9, 130.7, 147.0, 150.4.

#### 1,2-Bis((1-benzyl-1*H*-1,2,3-triazol-4-yl)methoxy) benzene (22c)

White solid (95%); ^1^H NMR (400 MHz, CDCl_3_) *δ* 5.15 (s, 4H), 5.44 (s, 4H), 6.85–6.90 (m, 2H), 6.95–7.00 (m, 2H), 7.19 (d, *J* = 1.9 Hz, 2H), 7.20 (d, *J* = 3.8 Hz, 2H), 7.30 (d, *J* = 1.9 Hz, 3H), 7.31 (d, *J* = 1.7 Hz, 2H), 7.58 (s, 2H); ^13^C NMR (100 MHz, CDCl_3_) *δ* 54.1, 63.5, 115.7, 122.2, 128.0, 128.7, 129.0, 134.5, 148.4.

#### 1,2-Bis((1-(2-methylbenzyl)-1*H*-1,2,3-triazol-4-yl)methoxy) benzene (23c)

White solid (94%); ^1^H NMR (400 MHz, CDCl_3_) *δ* 2.21 (s, 6H), 5.12 (s, 4H), 5.43 (s, 4H), 6.83–6.87 (m, 2H), 6.93–6.98 (m, 2H), 7.03 (d, *J* = 7.6 Hz, 2H), 7.11–7.16 (m, 4H), 7.20–7.24 (m, 2H), 7.47 (bs, 2H); ^13^C NMR (100 MHz, CDCl_3_) *δ* 18.8, 63.4, 115.6, 122.0, 126.4, 128.9, 129.1, 130.8, 132.4, 136.6, 148.3.

#### 1,2-Bis((1-(3-methoxybenzyl)-1*H*-1,2,3-triazol-4-yl)methoxy) benzene (24c)

White solid (94%); ^1^H NMR (400 MHz, CDCl_3_) *δ* 3.72 (s, 6H), 5.12 (s, 4H), 5.41 (s, 4H), 6.74 (s, 2H), 6.78 (m, 4H), 6.88 (bs, 2H), 6.98 (bs, 4H), 7.22 (t, *J* = 8.4 Hz, 2H), 7.71 (bs, 2H); ^13^C NMR (100 MHz, CDCl_3_) *δ* 54.3, 55.1, 63.5, 113.5, 113.9, 115.4, 120.1, 121.9, 129.9, 135.7, 148.2, 159.7.

#### 1,2-Bis((1-(4-bromobenzyl)-1*H*-1,2,3-triazol-4-yl)methoxy) benzene (25c)

Yellow solid (96%); ^1^H NMR (400 MHz, CDCl_3_) *δ* 5.16 (s, 4H), 5.45 (s, 4H), 6.90–6.93 (m, 2H), 6.99–7.02 (m, 2H), 7.09 (d, *J* = 8.7 Hz, 4H), 7.44 (d, *J* = 7.7 Hz, 4H), 7.70 (bs, 2H); ^13^C NMR (100 MHz, CDCl_3_) *δ* 53.5, 115.6, 122.2, 122.7, 129.6, 132.1, 133.5, 148.4.

#### 1,3-Bis((1-benzyl-1*H*-1,2,3-triazol-4-yl)methoxy) benzene (26c)

Yellow solid (94%) yield; ^1^H NMR (400 MHz, CDCl_3_) *δ* 5.11 (s, 4H), 5.61 (s, 4H), 6.61 (d, *J* = 2.2 Hz, 1H), 6.63 (d, *J* = 2.2 Hz, 1H), 6.71 (t, *J* = 2.3 Hz, 1H), 7.18 (t, *J* = 8.2 Hz, 1H), 7.30–7.40 (m, 10H), 8.28 (s, 2H);^13^C NMR (100 MHz, CDCl_3_) *δ* 52.8, 61.1, 101.6, 107.3, 124.6, 127.9, 128.1, 128.7, 135.9, 159.2.

#### 1,3-Bis((1-(2-methylbenzyl)-1*H*-1,2,3-triazol-4-yl)methoxy) benzene (27c)

White solid (92%); ^1^H NMR (400 MHz, CDCl_3_) *δ* 2.24 (s, 6H), 5.08 (s, 4H), 5.49 (4H), 6.52–6.55 (m, 3H), 7.09–7.13 (m, 3H), 7.16–7.20 (m, 4H), 7.23–7.27 (m, 2H), 7.40 (s, 2H); ^13^C NMR (100 MHz, CDCl_3_) *δ* 18.9, 52.3, 62.0, 102.1, 107.5, 126.6, 129.1, 129.4, 129.9, 130.9, 132.3, 136.8, 159.3.

#### 1,3-Bis((1-(3-methoxybenzyl)-1*H*-1,2,3-triazol-4-yl)methoxy) benzene (28c)

White solid (91%); ^1^H NMR (400 MHz, CDCl_3_) *δ* 3.73 (s, 6H), 5.11 (s, 4H), 5.46 (s, 4H), 6.54 (m, 3H), 6.76 (s, 2H), 6.83 (t, *J* = 7.5 Hz, 1H), 7.25 (m, 2H) 7.58 (bs, 2H); ^13^C NMR (100 MHz, CDCl_3_) *δ* 54.1, 55.2, 61.8, 101.9, 107.5, 113.6, 114.1, 120.1, 129.9, 130.1, 135.8, 159.3, 159.9.

#### 1,3-Bis((1-(4-bromobenzyl)-1*H*-1,2,3-triazol-4-yl)methoxy) benzene (29c)

Yellow solid (96%); ^1^H NMR (400 MHz, DMSO-d_6_) *δ* 5.07 (s, 4H), 5.56 (s, 4H), 6.57 (d, *J* = 2.3 Hz, 1H), 6.59 (d, *J* = 2.3 Hz, 1H), 6.67 (t, *J* = 2.5 Hz, 1H), 7.14 (t, *J* = 7.9 Hz, 1H), 7.22–7.26 (m, 4H), 7.51–7.55 (m, 4H), 8.28 (s, 2H); ^13^C NMR (100 MHz, DMSO-d_6_) *δ* 52.5, 61.5, 102.1, 107.8, 121.9, 125.2, 130.6, 132.1, 135.8, 143.5, 159.7.

#### 1,4-Bis((1-benzyl-1*H*-1,2,3-triazol-4-yl)methoxy) benzene (30c)

Yellow solid (92%); ^1^H NMR (400 MHz, CDCl_3_) *δ* 5.06 (s, 4H), 5.60 (s, 4H), 6.95 (s, 4H), 7.30 (m, 2H), 7.32–7.33 (m, 3H), 7.34 (d, *J* = 1.8 Hz, 1H), 7.35–7.36 (m, 2H), 7.37–7.40 (m, 2H), 8.25 (s, 2H); ^13^C NMR (100 MHz, CDCl_3_) *δ* 52.8, 61.6, 115.6, 124.5, 127.9, 128.1, 128.73, 135.9, 143.2, 152.3.

#### 1,4-Bis((1-(2-methylbenzyl)-1*H*-1,2,3-triazol-4-yl)methoxy) benzene (31c)

White solid (93%); ^1^H NMR (400 MHz, CDCl_3_) *δ* 2.24 (s, 6H), 5.06 (s, 4H), 5.49 (s, 4H), 6.83 (s, 4H), 7.10–7.11 (m, 2H), 7.16–7.19 (m, 4H), 7.23–7.27 (m, 2H), 7.41 (bs, 2H); ^13^C NMR (100 MHz, CDCl_3_) *δ* 18.9, 52.4, 62.6, 115.8, 126.6, 129.1, 129.3, 130.9, 132.3, 136.8, 152.7.

#### 1,4-Bis((1-(3-methoxybenzyl)-1*H*-1,2,3-triazol-4-yl)methoxy) benzene (32c)

White solid (92%); ^1^H NMR (400 MHz, CDCl_3_) *δ* 3.74 (s, 6H), 5.09 (bs, 4H), 5.46 (s, 4H), 6.75 (s, 2H), 6.81–6.86 (m, 8H), 7.24 (m, 2H), 7.67 (bs, 2H); ^13^C NMR (100 MHz, CDCl_3_) *δ* 54.3, 55.2, 62.4, 113.6, 114.1, 115.7, 120.1, 130.0, 135.7, 152.6, 159.9.

#### 1,4-Bis((1-(4-bromobenzyl)-1*H*-1,2,3-triazol-4-yl)methoxy) benzene (33c)

Yellow solid (92%); ^1^H NMR (400 MHz, DMSO-d_6_) *δ* 5.07 (s, 4H), 5.60 (s, 4H), 6.95 (s, 4H), 7.26 (d, *J* = 8.6 Hz, 4H), 7.57 (d, *J* = 8.6 Hz, 4H), 8.26 (s, 2H); ^13^C NMR (100 MHz, DMSO-d_6_) *δ* 52.1, 61.5, 115.6, 121.4, 124.6, 130.2, 131.7, 135.4, 143.3, 152.3.

#### 1-Phenyl-2-(4-phenyl-1*H*-1,2,3-triazol-1-yl)ethan-1-one (35c)

Pale yellow solid (92%) yield; ^1^H NMR (400 MHz, DMSO-d_6_) *δ* 6.26 (s, 2H), 7.35 (m, 1H), 7.47 (t, *J* = 7.5 Hz, 2H), 7.63 (t, *J* = 7.5 Hz, 2H), 7.75 (m, 1H), 7.87 (d, *J* = 7.6 Hz, 2H), 8.09 (d, *J* = 7.8 Hz, 2H), 8.52 (s, 1H); ^13^C NMR (100 MHz, DMSO-d_6_) *δ* 56.0, 123.0, 125.2, 128.2, 127.9, 128.2, 128.9, 130.7, 134.3, 146.3, 192.2.

#### 1-(4-Methoxyphenyl)-2-(4-phenyl-1*H*-1,2,3-triazol-1-yl)ethan-1-one (36c)

Pale yellow solid (95%) yield; ^1^H NMR (400 MHz, CDCl_3_) *δ* 3.84 (s, 3H), 5.77 (s, 2H), 6.93 (d, *J* = 6.9 Hz, 2H), 7.28 (t, *J* = 7.3 Hz, 1H), 7.37 (t, *J* = 7.8 Hz, 2H), 7.90 (s, 1H), 7.92 (d, *J* = 7.8 Hz, 2H); ^13^C NMR (100 MHz, CDCl_3_) *δ* 55.2, 114.4, 125.8, 126.9, 128.8, 130.6, 164.6, 188.6.

## Conflicts of interest

There are no conflicts to declare.

## Supplementary Material

RA-009-C9RA00972H-s001
