# Supplementary material for: Synthesis of a novel resorcin[4]arene–glucose conjugate and its catalysis of the CuAAC reaction for the synthesis of 1,4-disubstituted 1,2,3-triazoles in water
Source: RSC Adv. 2019 Mar 29;9(18):10109–16. doi: 10.1039/c9ra00972h (PMC9062644; doi:10.1039/c9ra00972h)
Supplement: RA-009-C9RA00972H-s001 [file RA-009-C9RA00972H-s001.pdf]

Electronic Supplementary Material (ESI) for RSC Advances.
This journal is © The Royal Society of Chemistry 2019

Supporting Information

Synthesis of a novel resorcin[4]arene-glucose conjugate and its catalysis of the CuAAC reaction for the synthesis of 1,4-disubstituted 1,2,3-triazoles in water

Ali A. Husain and Kirpal S. Bisht*

Department of Chemistry, University of South Florida, 4202 East Fowler Avenue, Tampa, Florida 33620, USA.

kbisht@usf.edu

Table of Contents

General Information……………………………………………………………………………2

NMR data of new compounds **2**, **4**, **RG**, **6C**, **7c**, **10c**, **11c**, **19c**, **20c**, **22c**, **23c**, **24c**, **25c**, **26c**, **27c**, **28c**, **29c**, **30c**, **31c**, **32c**, **33c**, **35c**, **36c**, …..……….…………………………..……….…….3-23

General information

^{1}H-NMR spectra for screening the conversion (%) were recorded on a Bruker DPX-250. ^{1}H- and ^{13}C-NMR spectra were recorded on a Inova-400 spectrometers. Sample concentrations were about 10% (w/v) in $CDCl_3$ and DMSO-d_6.

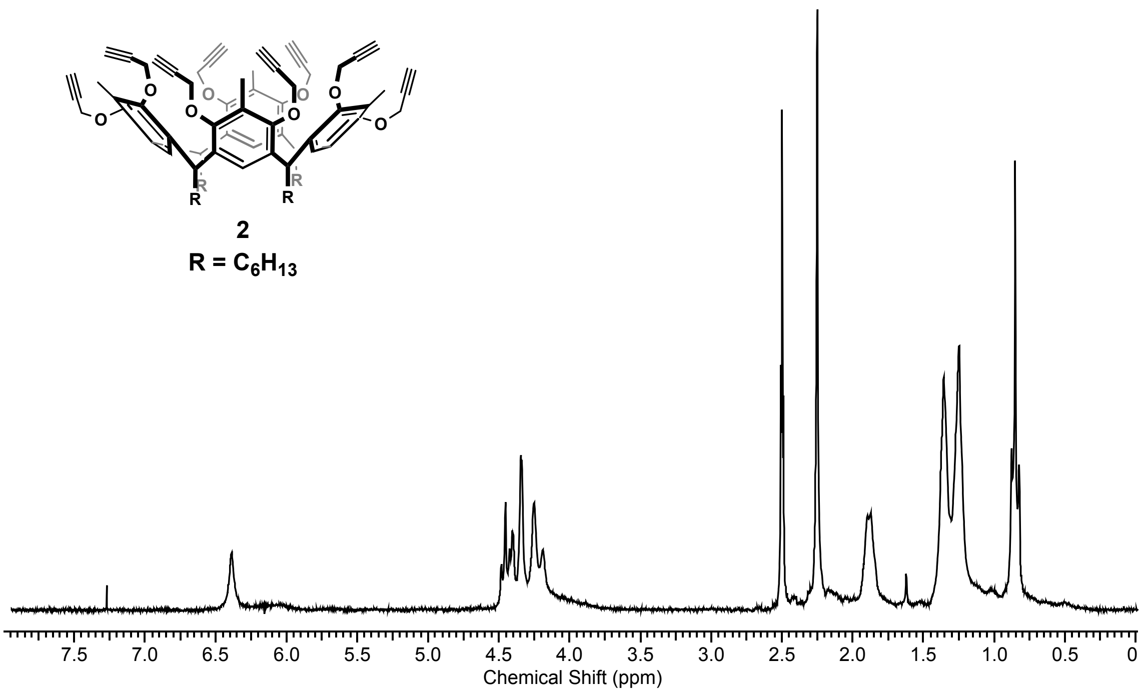

^{1}H-NMR spectrum of **2**.

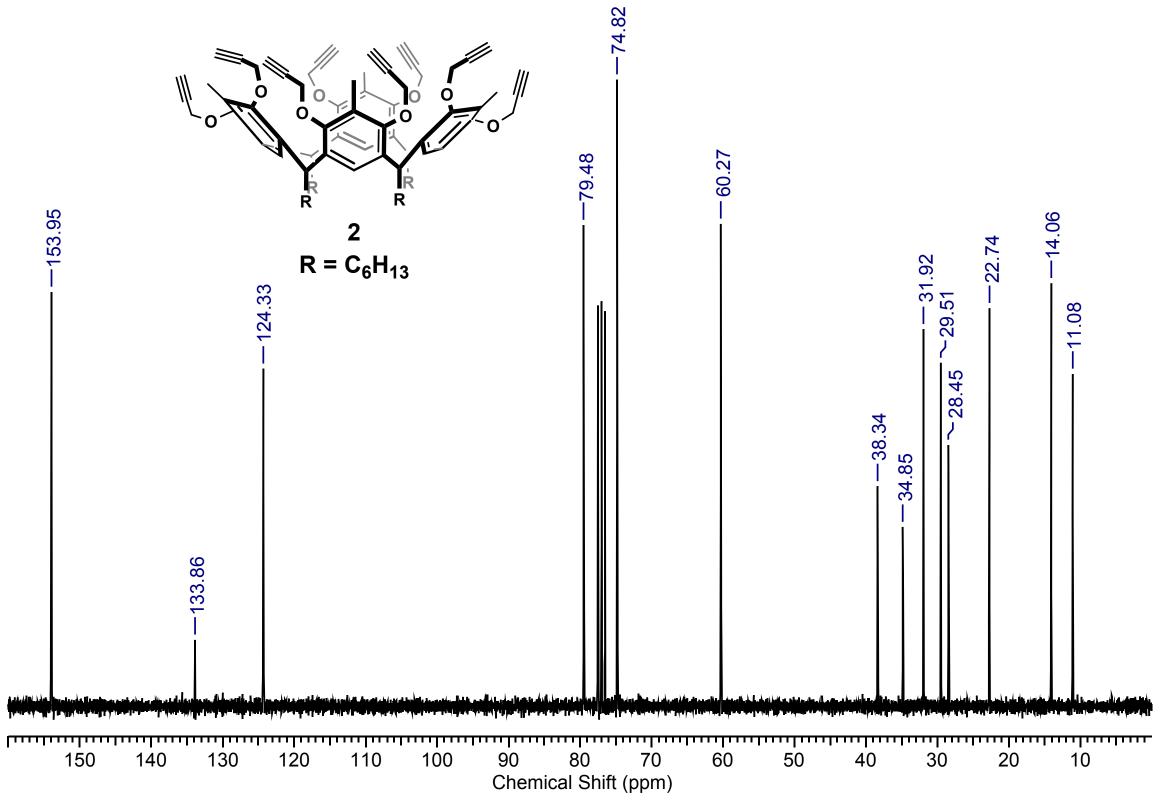

^{13}C-NMR spectrum of **2**.

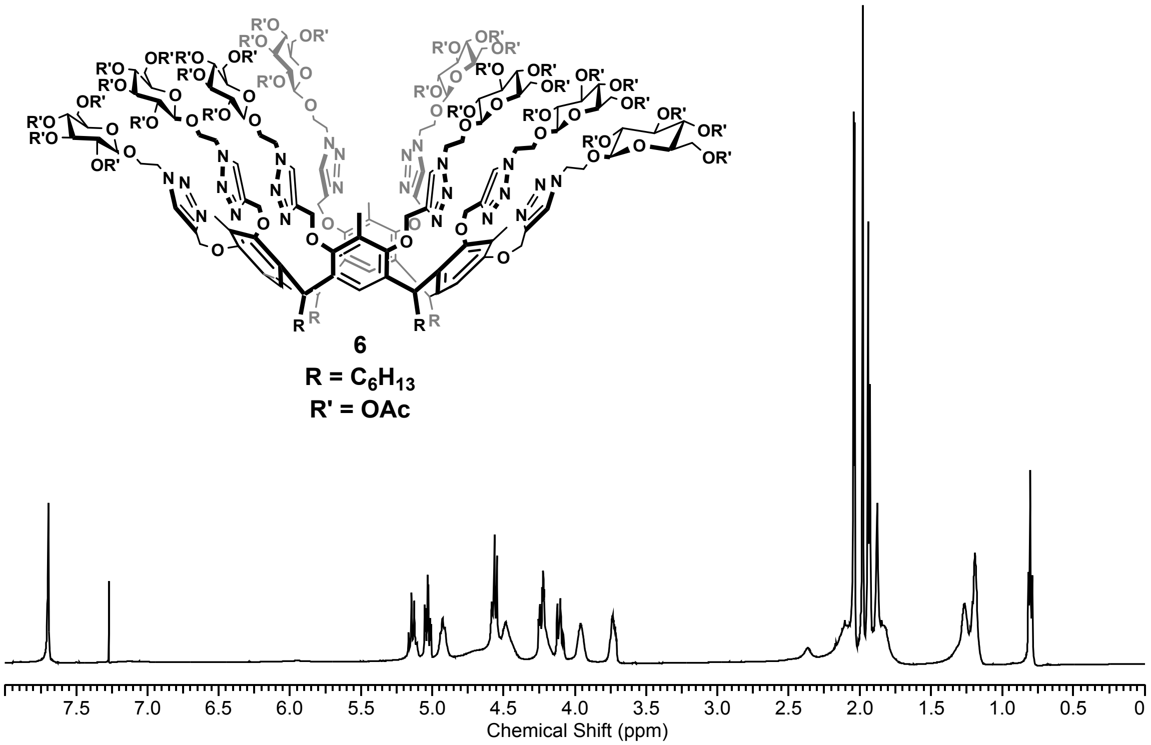

^{1}H-NMR spectrum of **6**.

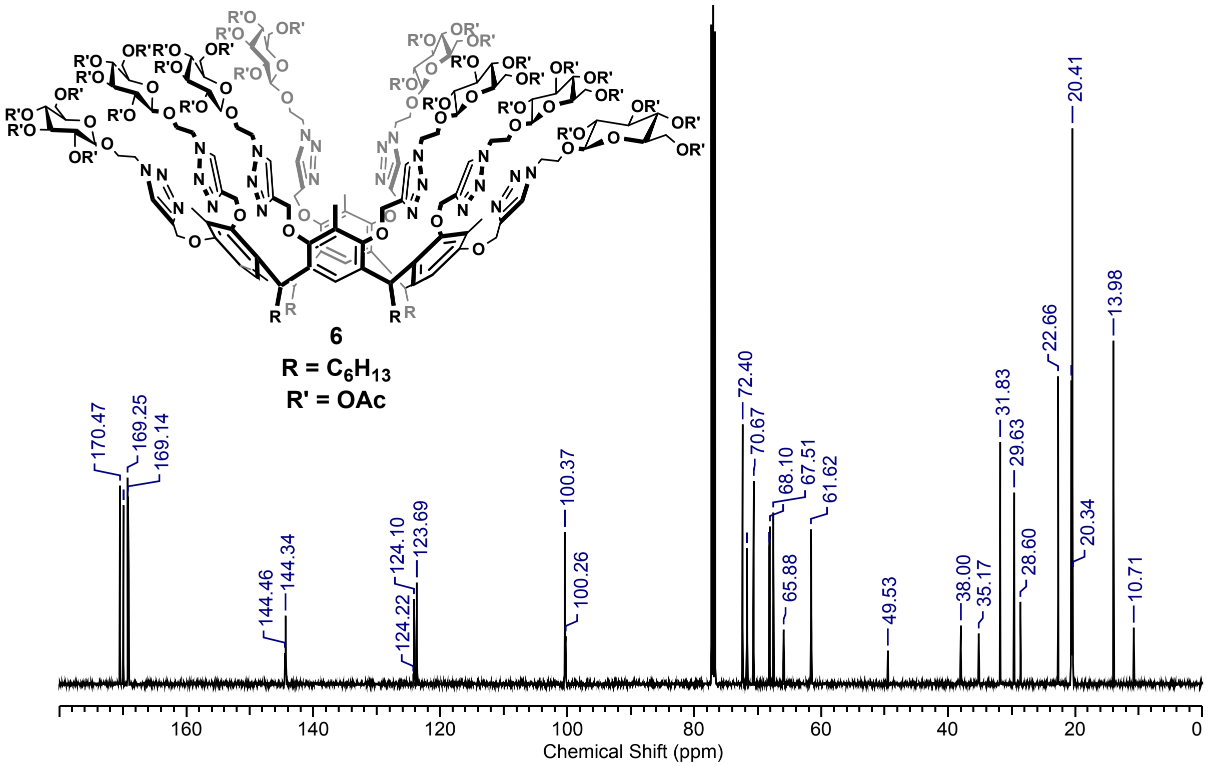

^{13}C-NMR spectrum of **6**.

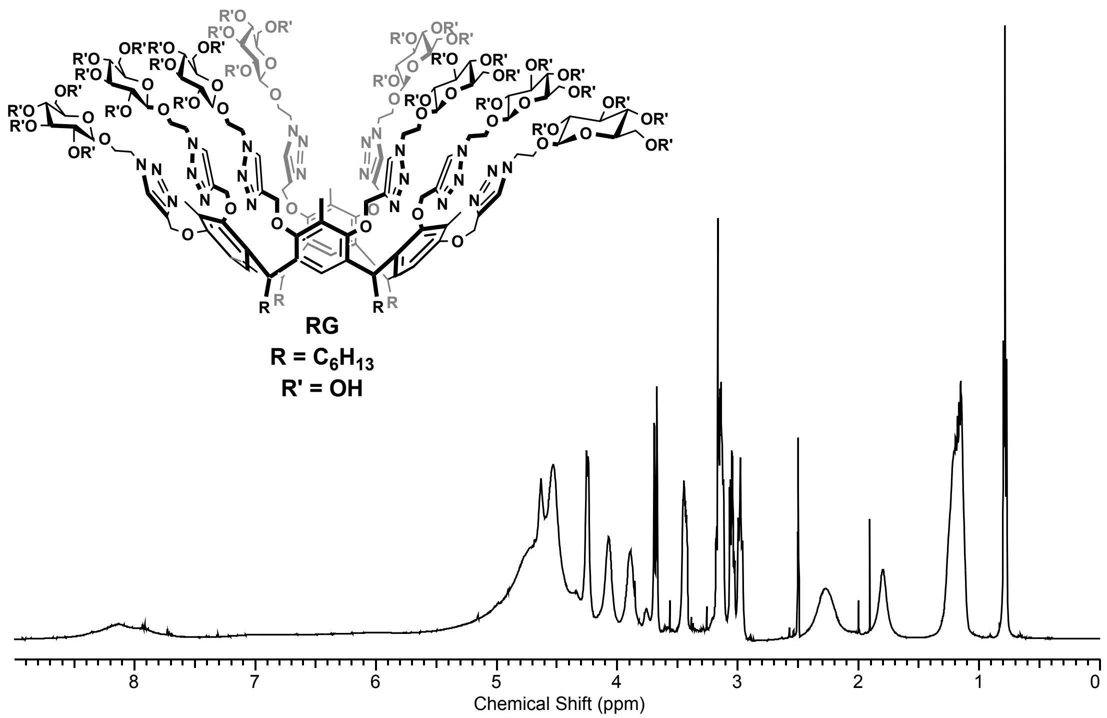

^{1}H-NMR spectrum of **RG**.

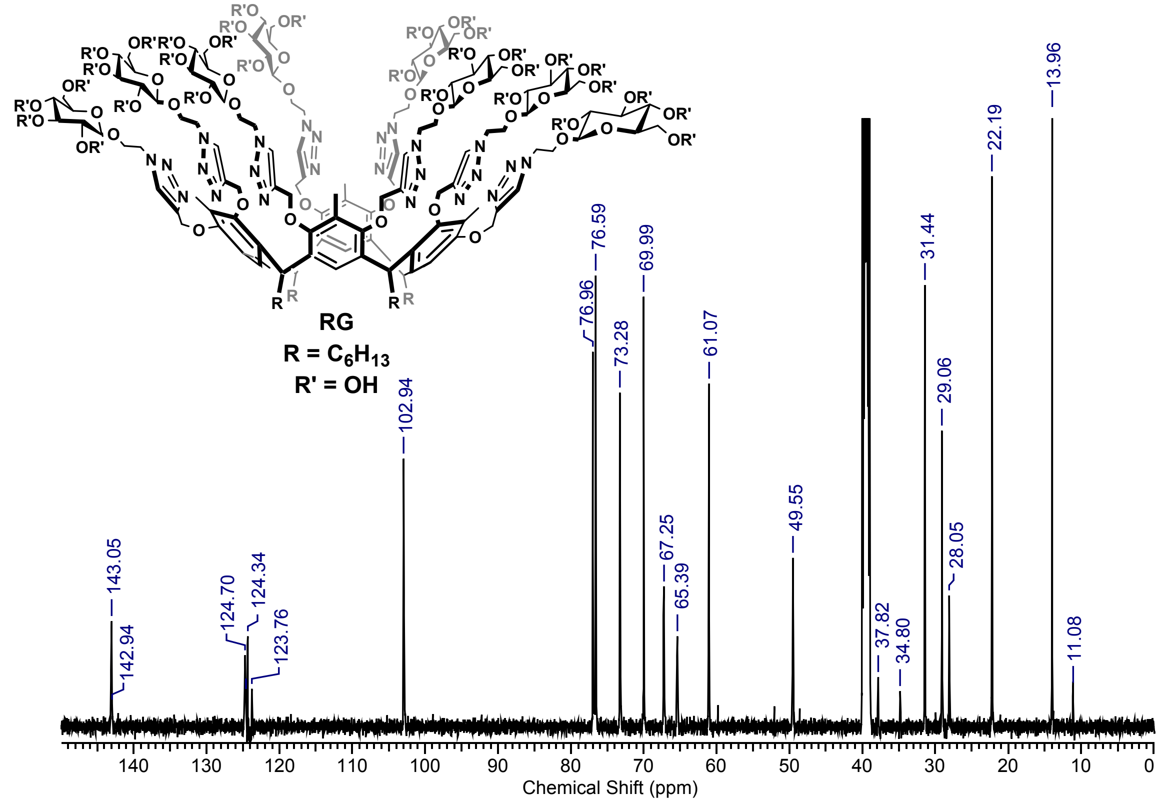

^{13}C-NMR spectrum of **RG**.

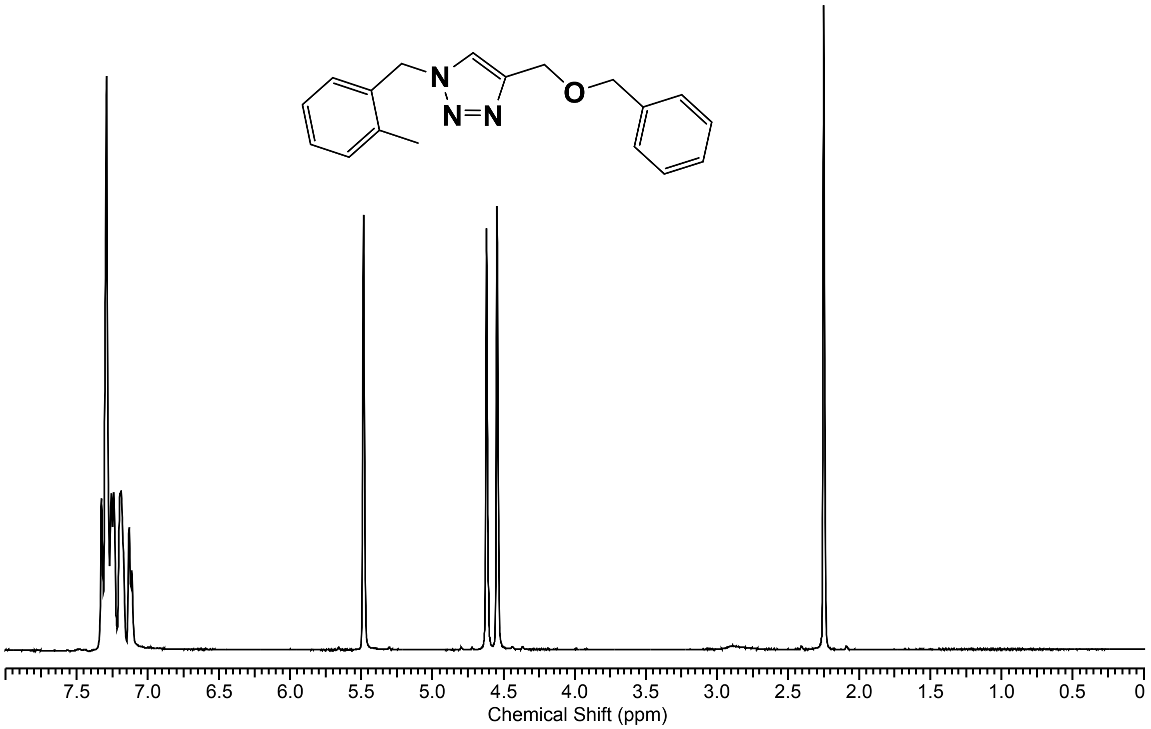

^{1}H-NMR spectrum of **6c**.

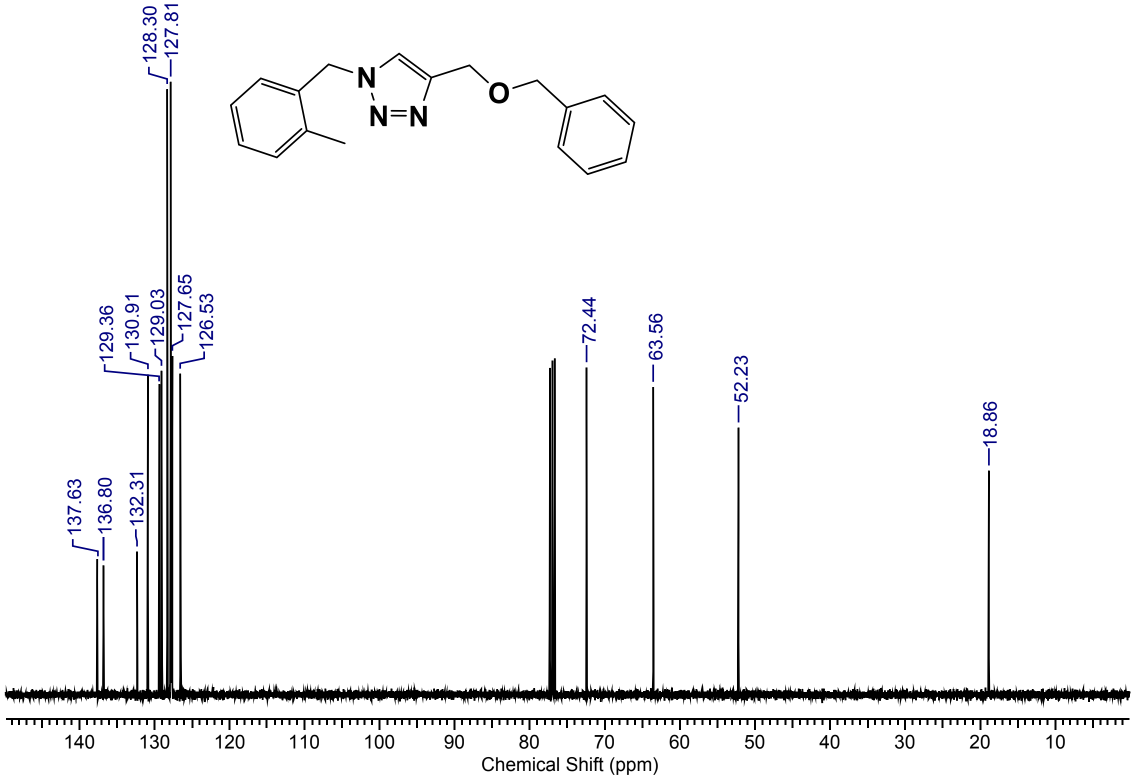

^{13}C-NMR spectrum of **6c**.

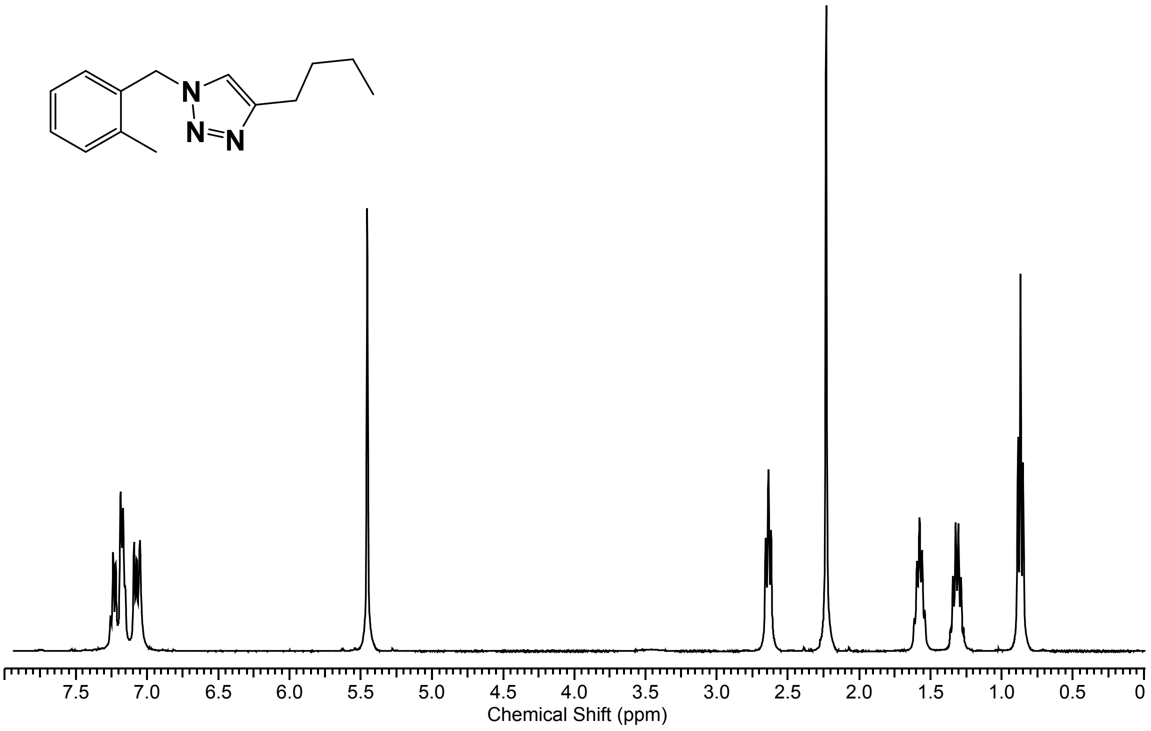

^{1}H-NMR spectrum of **7c**.

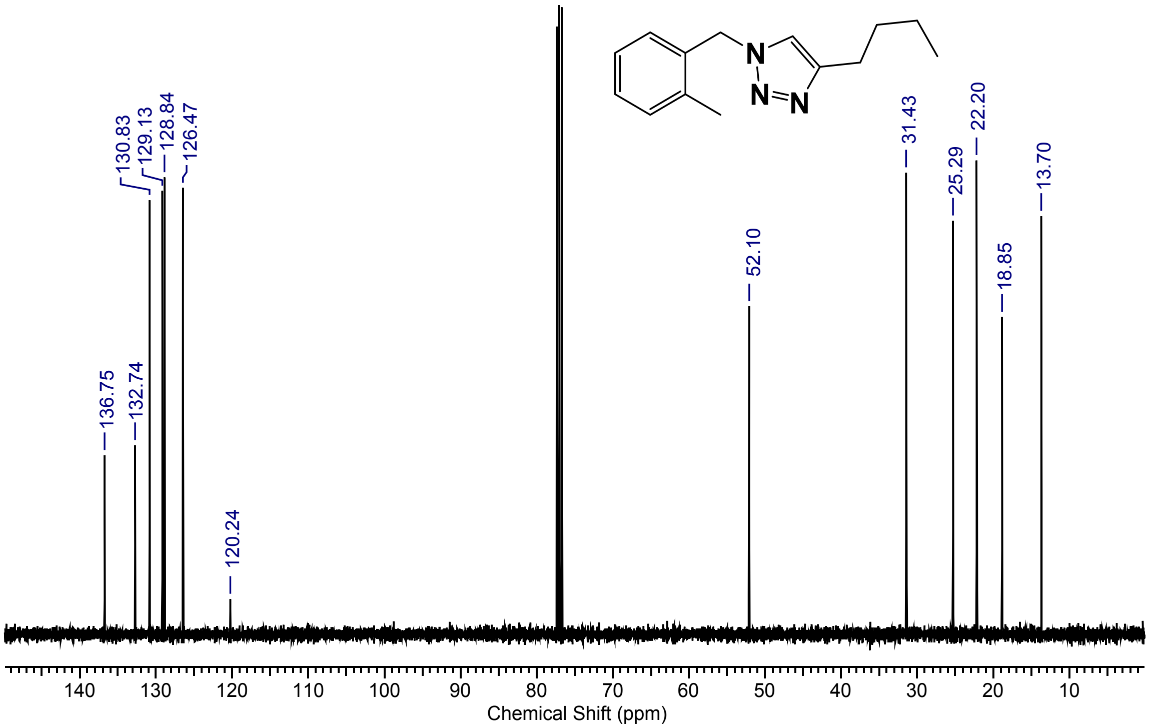

^{13}C-NMR spectrum of **7c**.

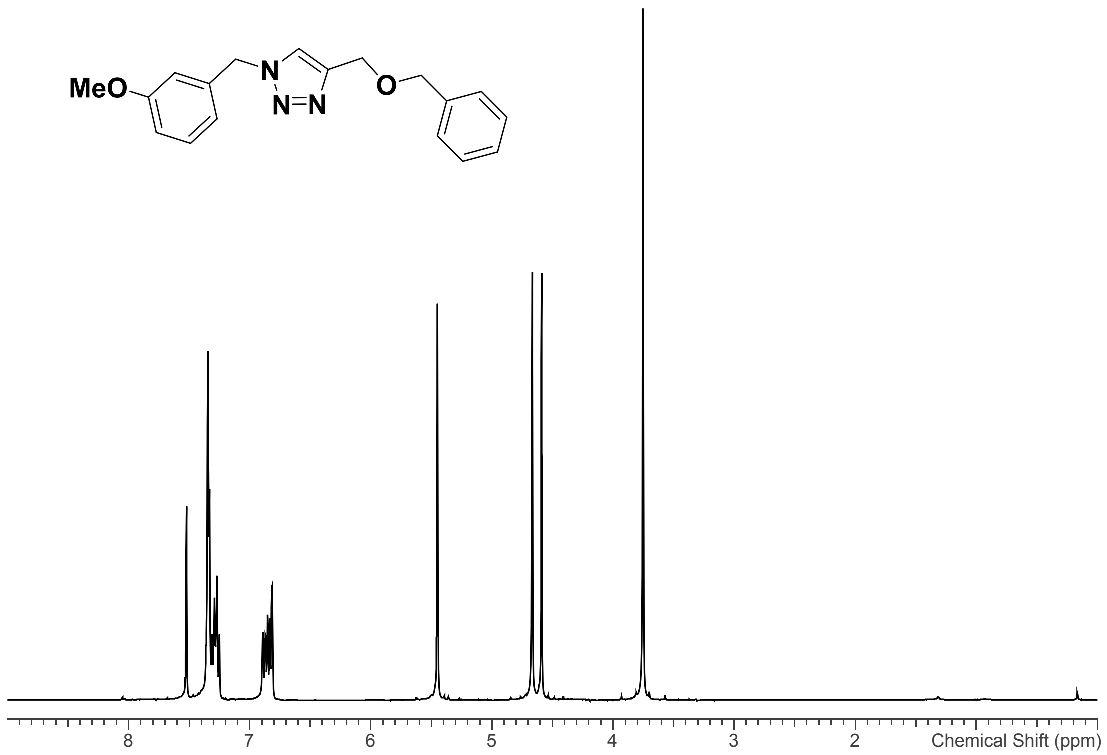

^{1}H-NMR spectrum of **10c**.

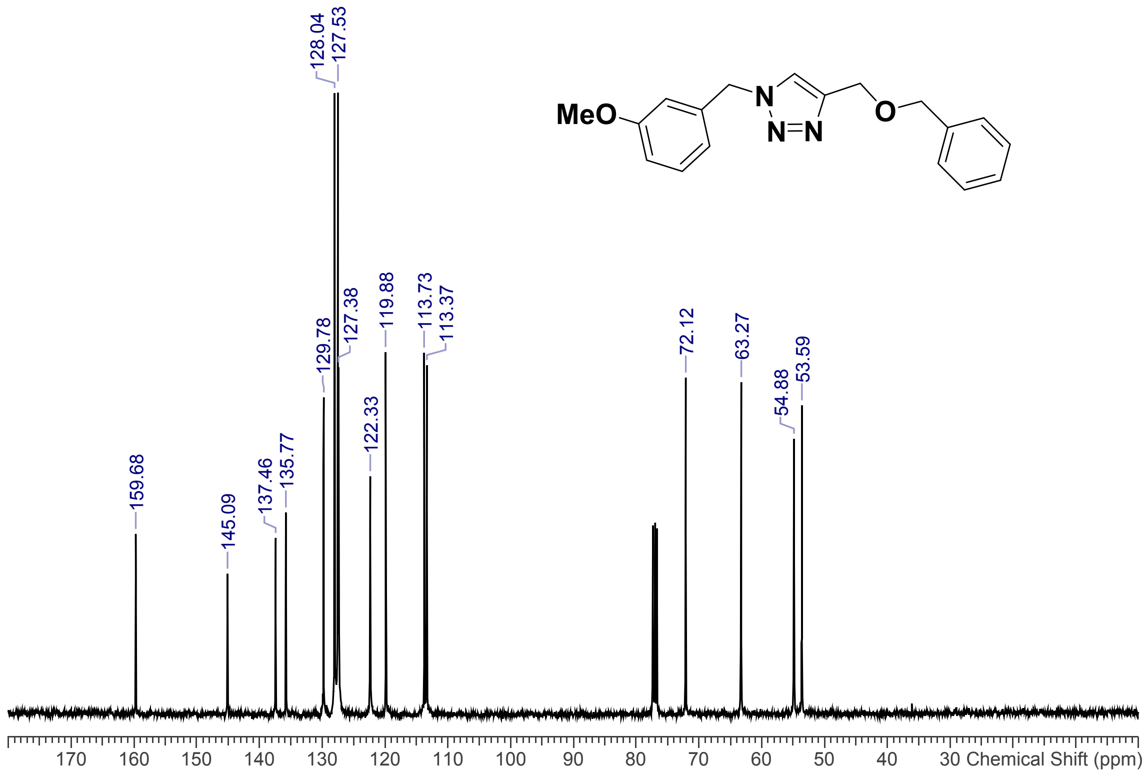

^{13}C-NMR spectrum of **10c**.

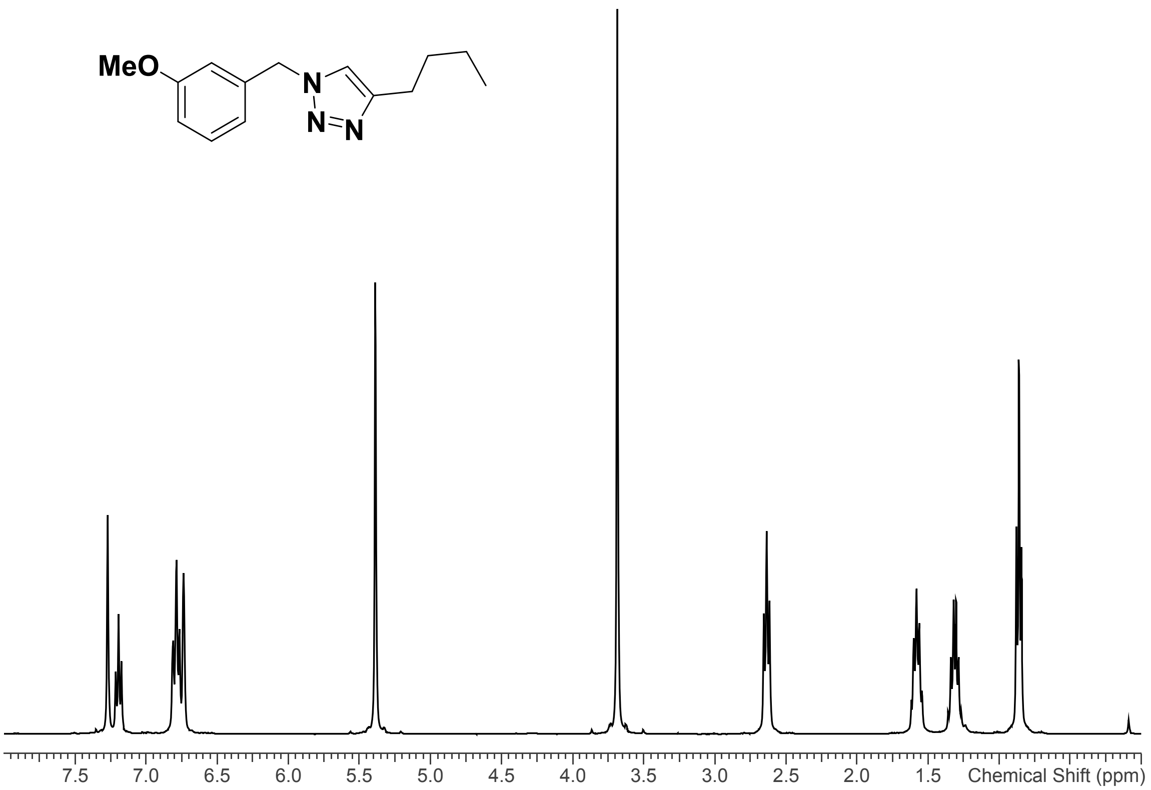

^{1}H-NMR spectrum of **triazole 11c**.

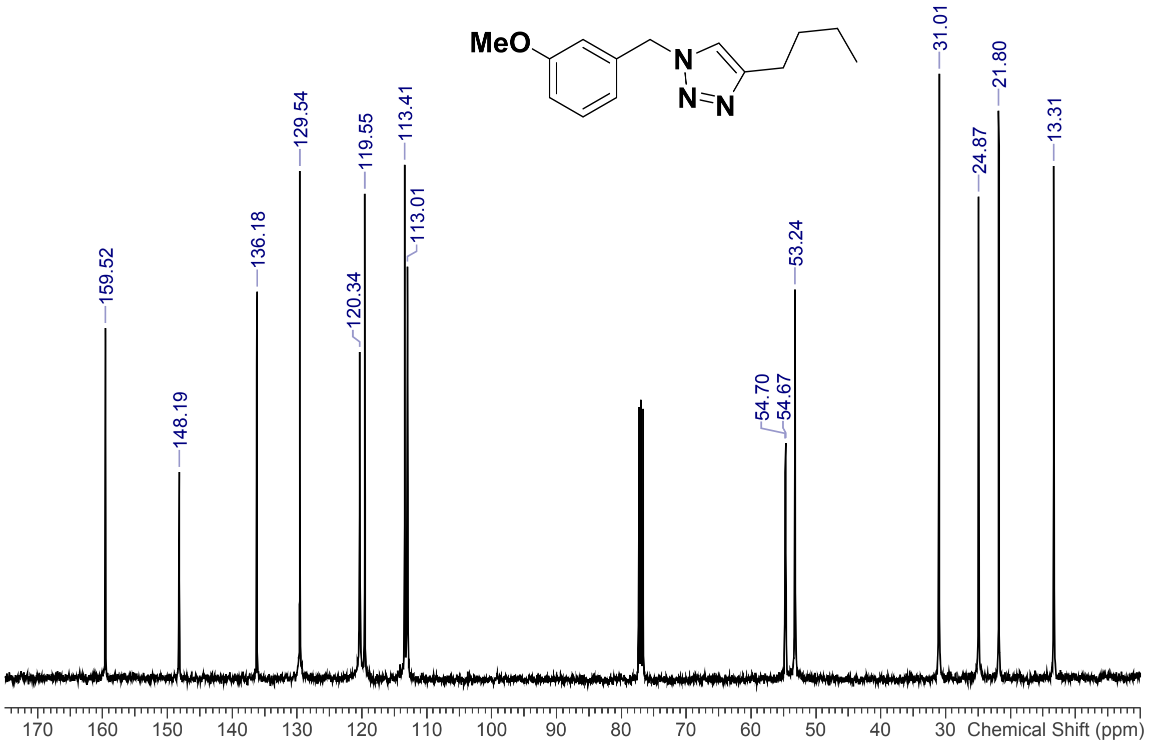

^{13}C-NMR spectrum of **triazole 11c**.

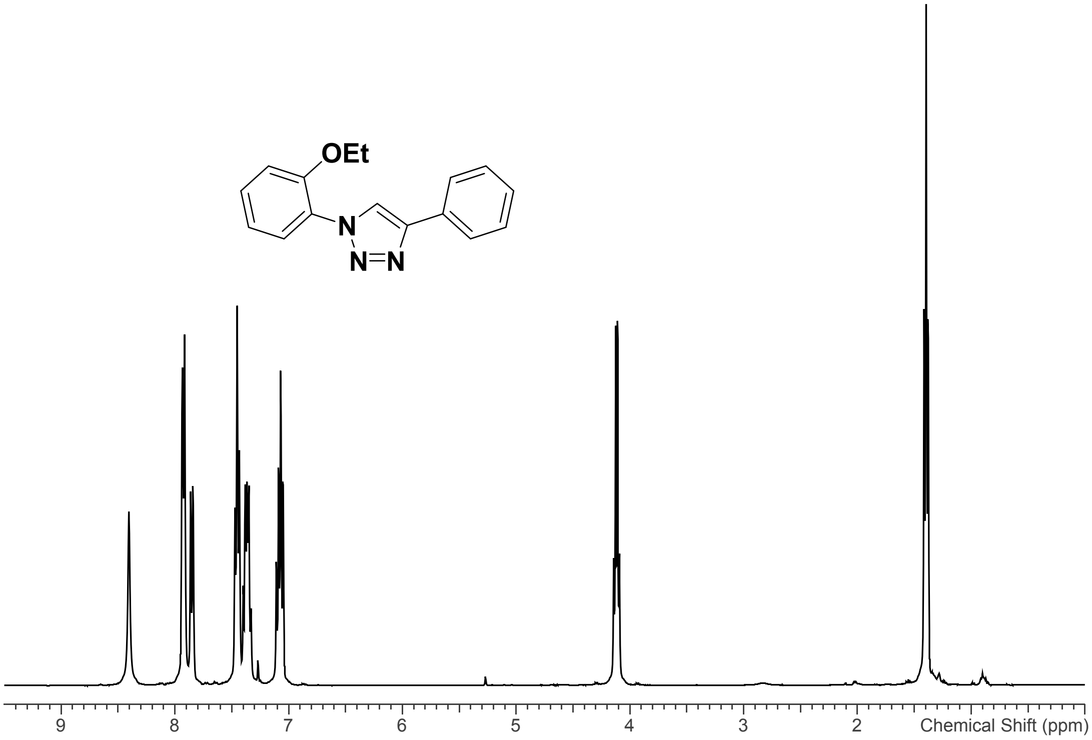

^{1}H-NMR spectrum of **19c**.

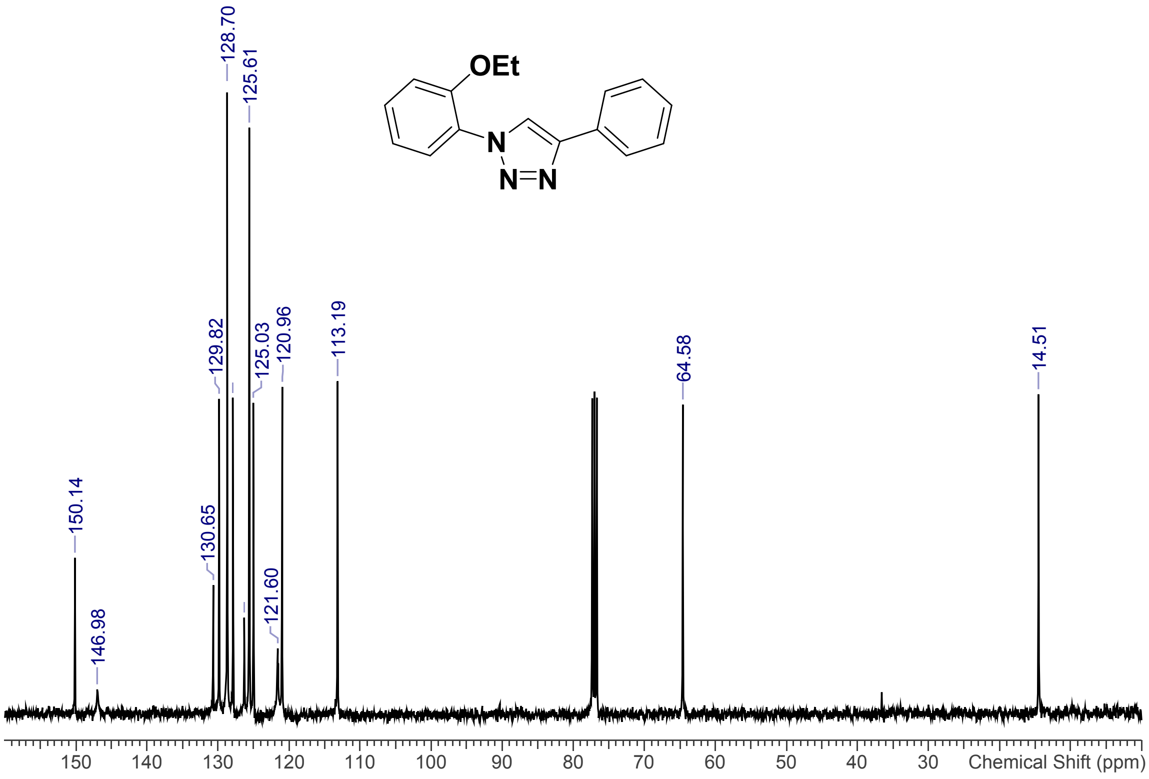

^{13}C-NMR spectrum of **19c**.

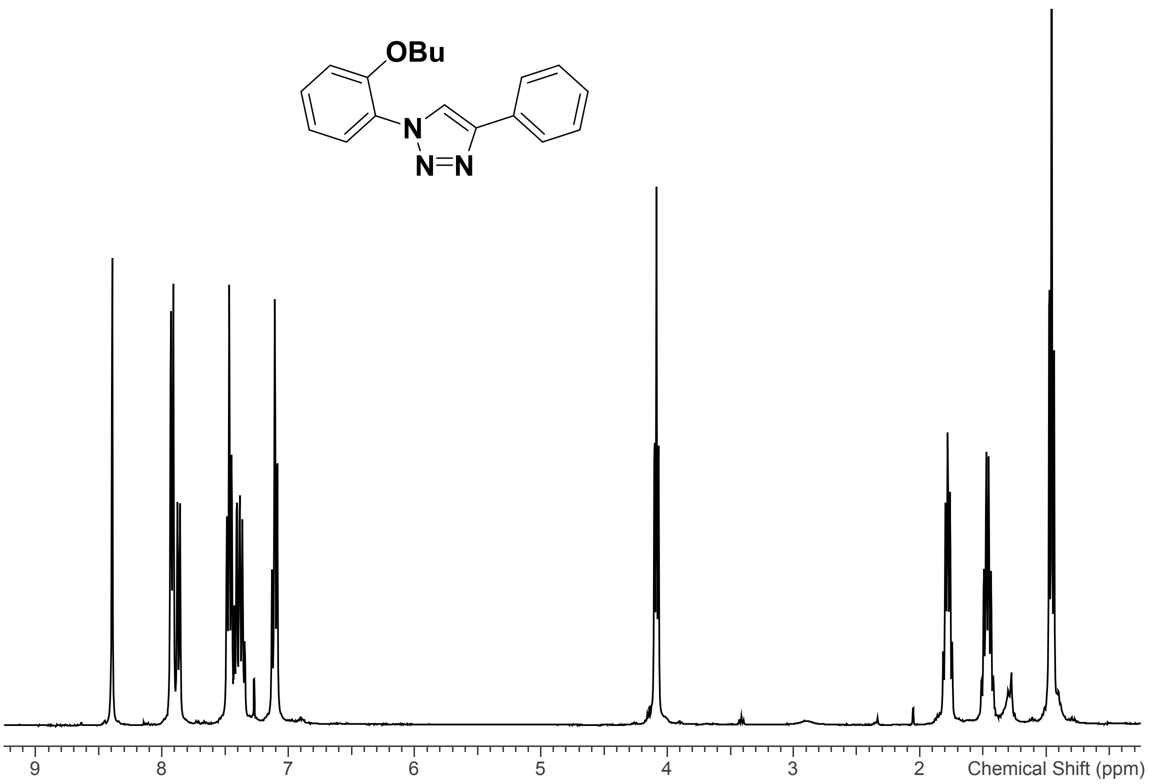

^{1}H-NMR spectrum of **20c**.

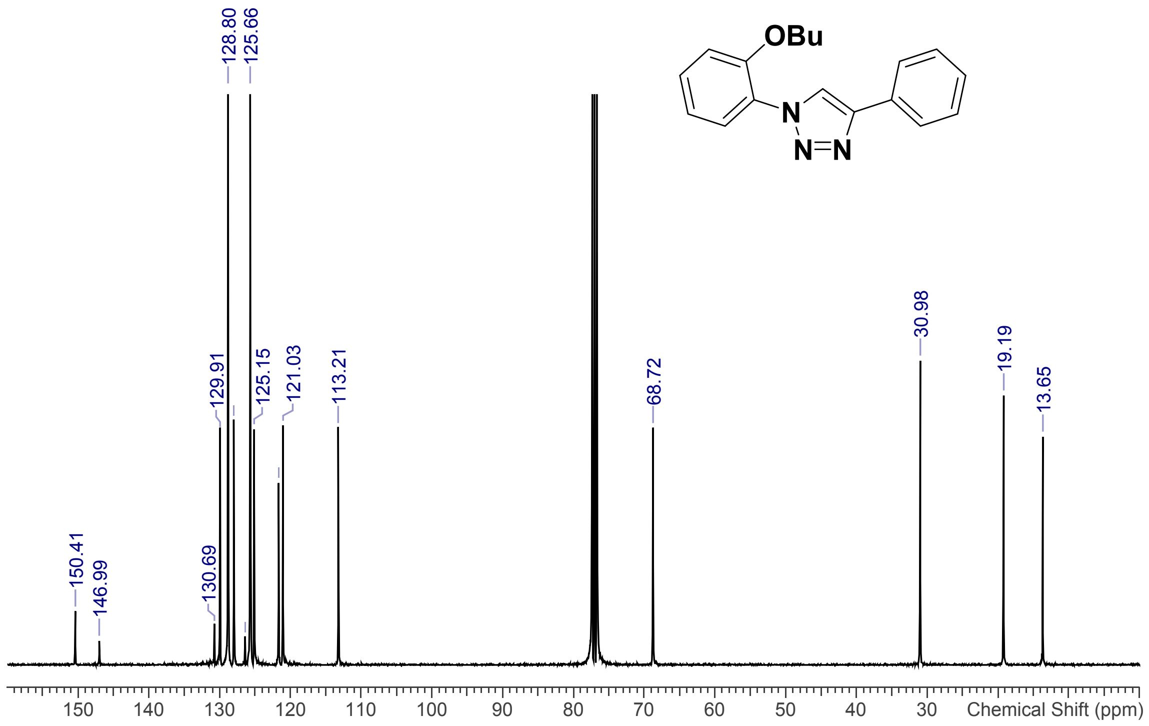

^{13}C-NMR spectrum of **20c**.

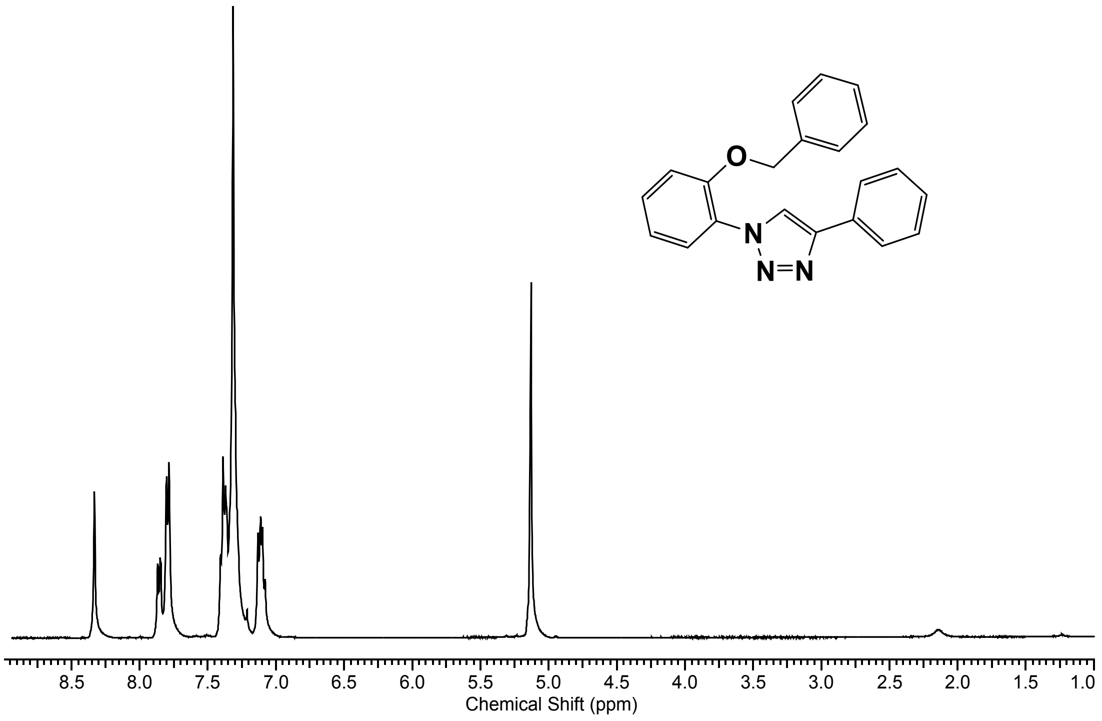

^{1}H-NMR spectrum of **21c**.

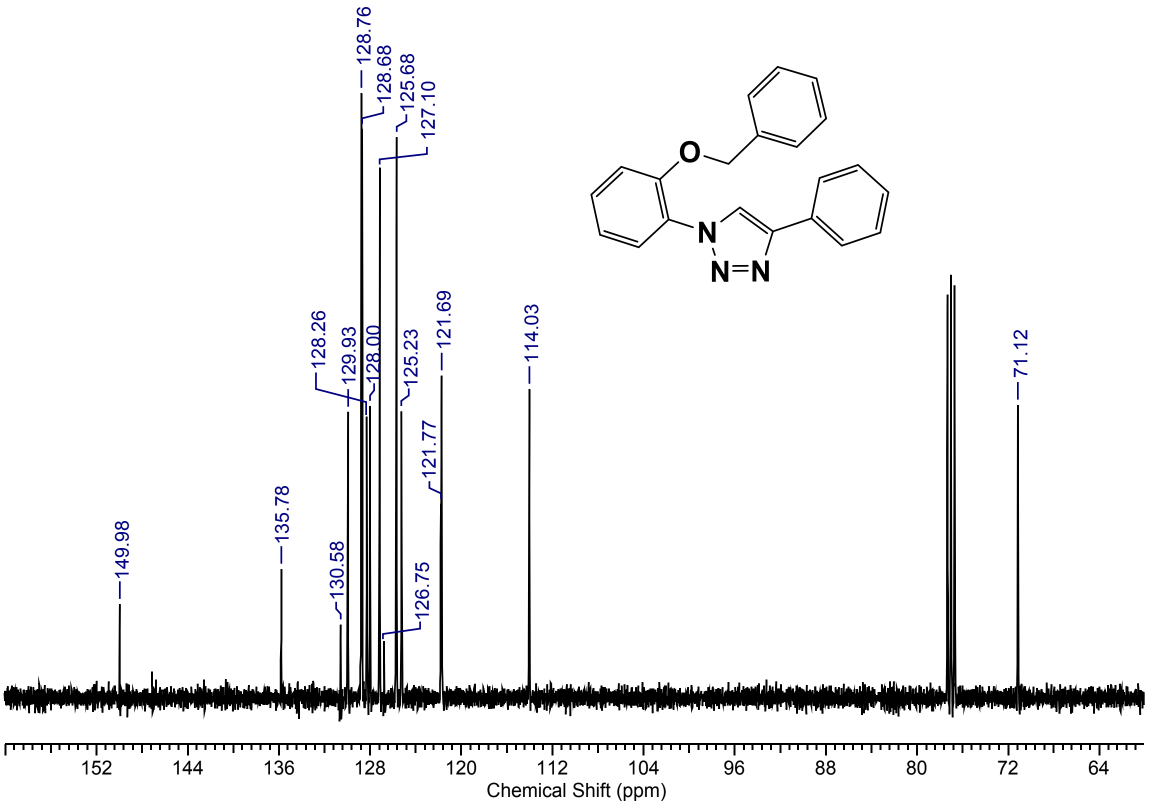

^{13}C-NMR spectrum of **21c**.

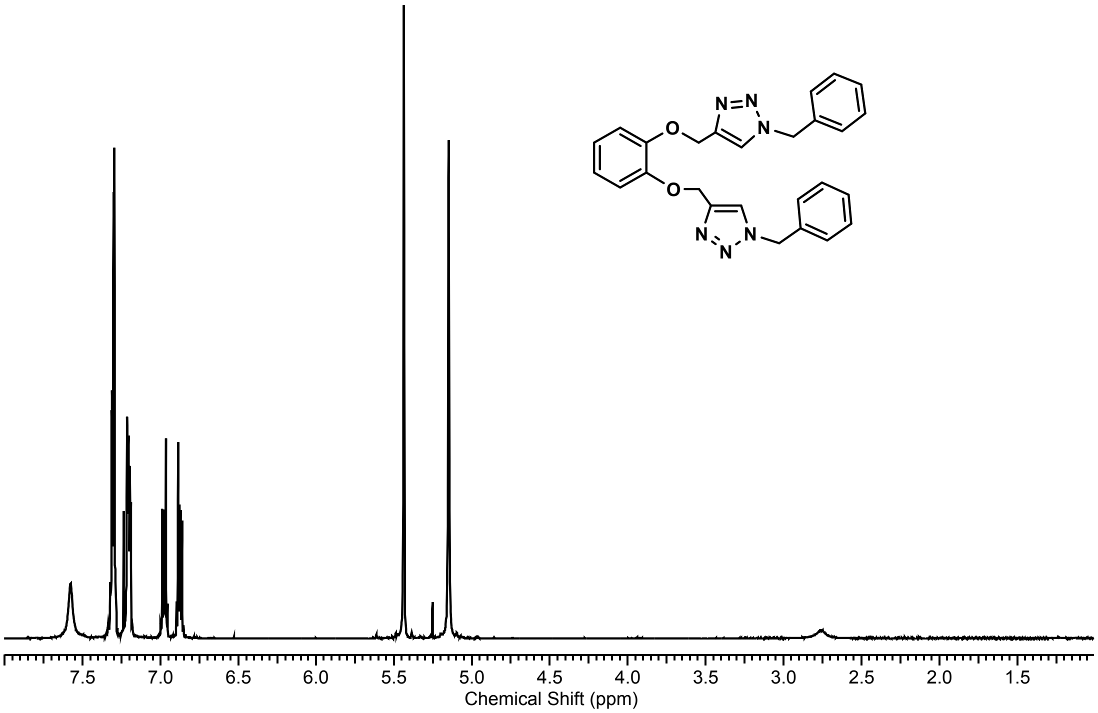

^{1}H-NMR spectrum of **22c**.

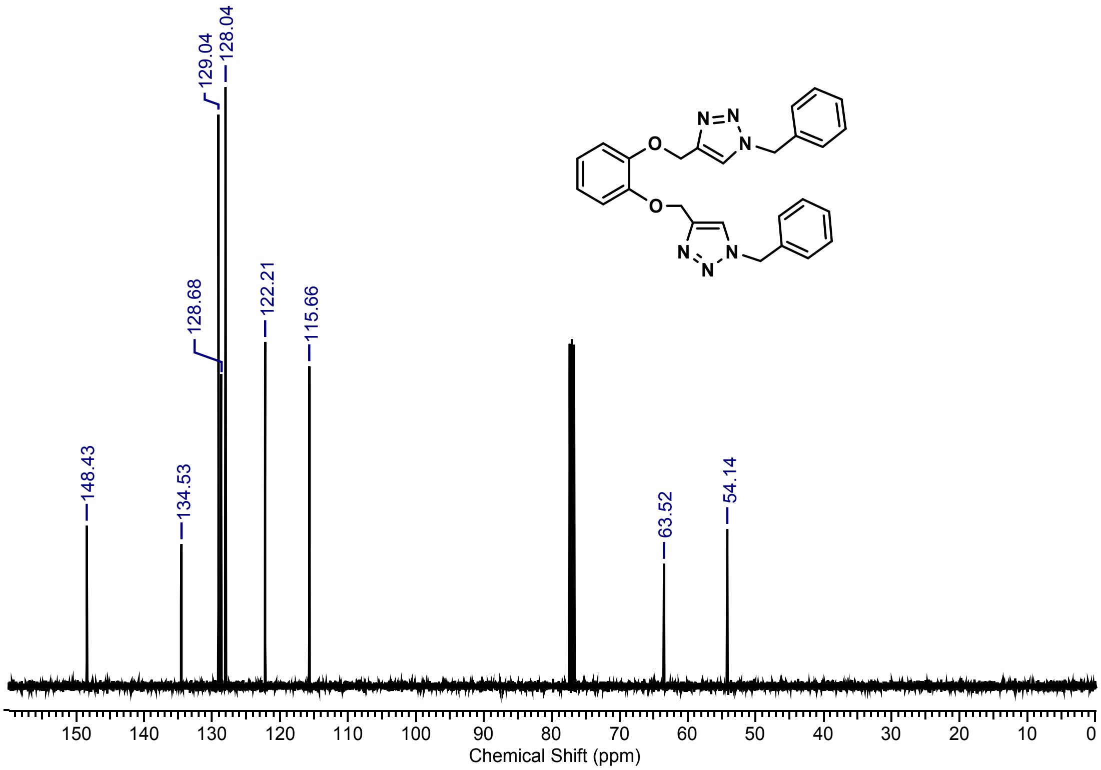

^{13}C-NMR spectrum of **22c**.

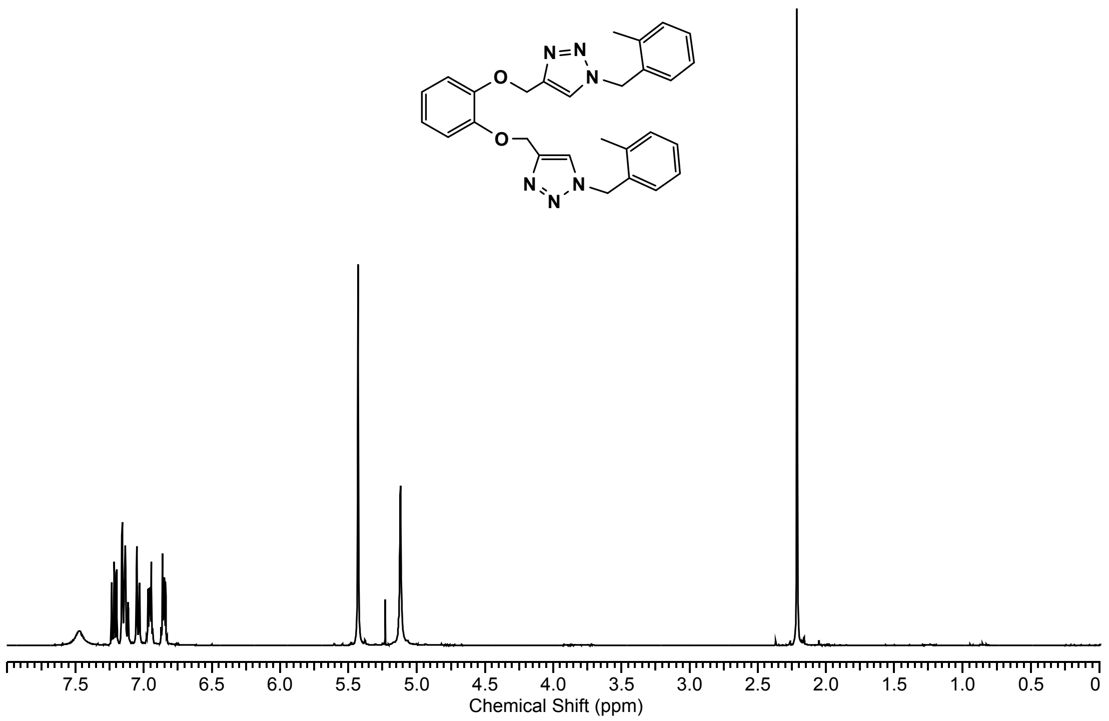

^{1}H-NMR spectrum of **23c**.

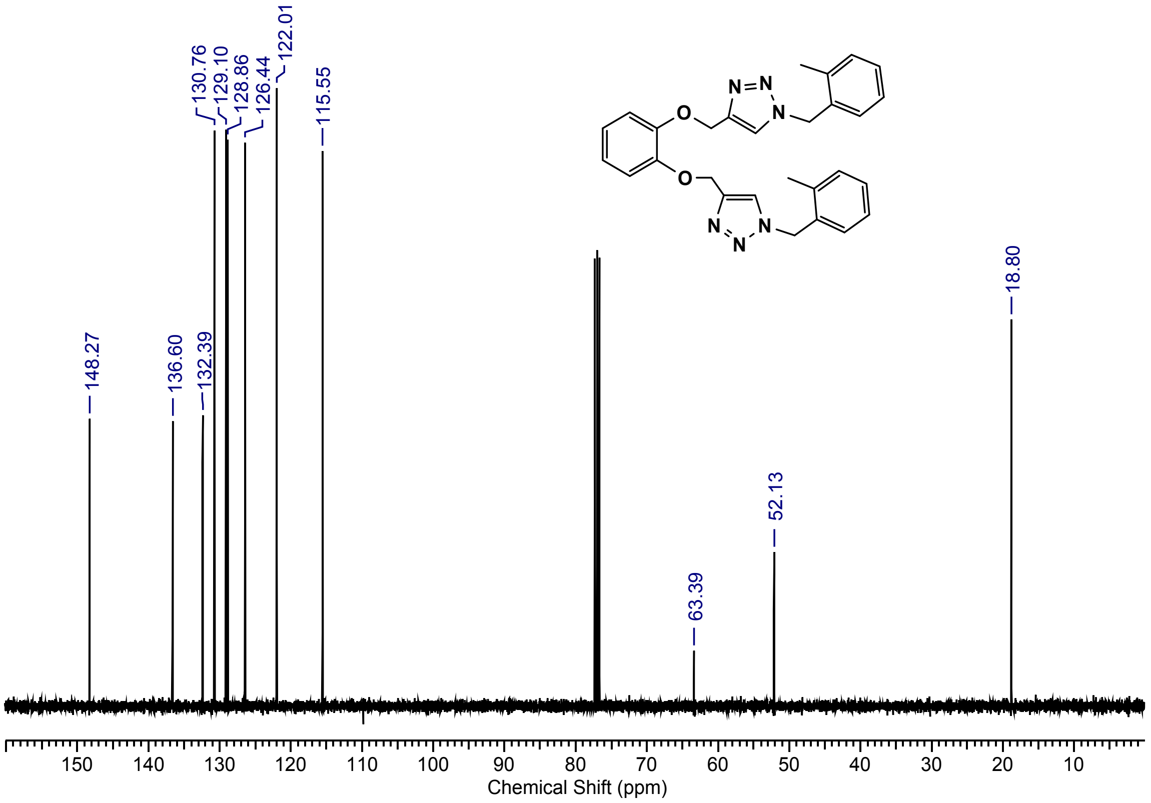

^{13}C-NMR spectrum of **23c**.

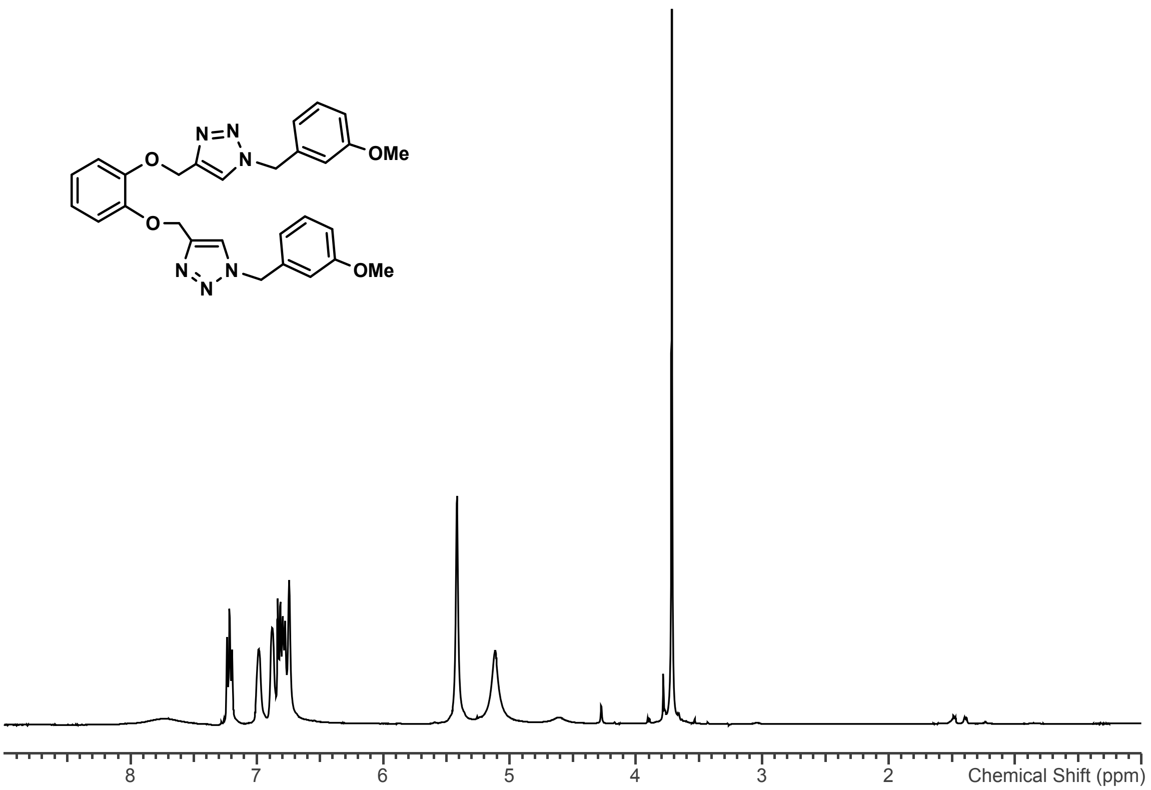

^{1}H-NMR spectrum of **24c**.

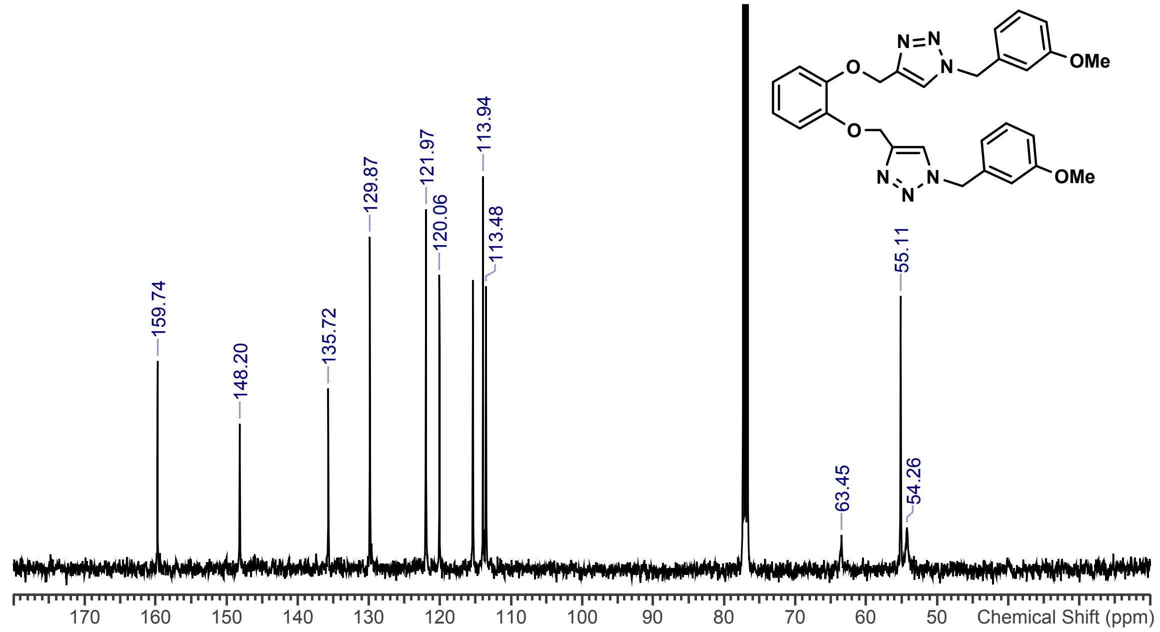

^{13}C-NMR spectrum of **24c**.

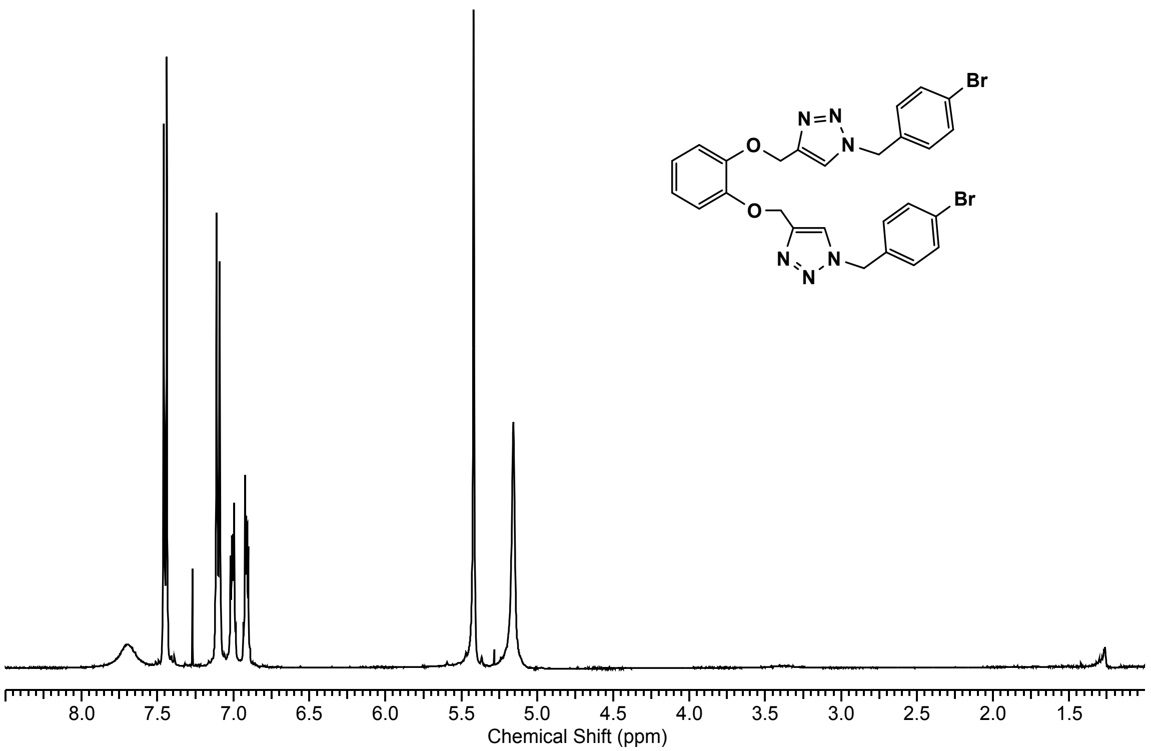

^{1}H-NMR spectrum of **25c**.

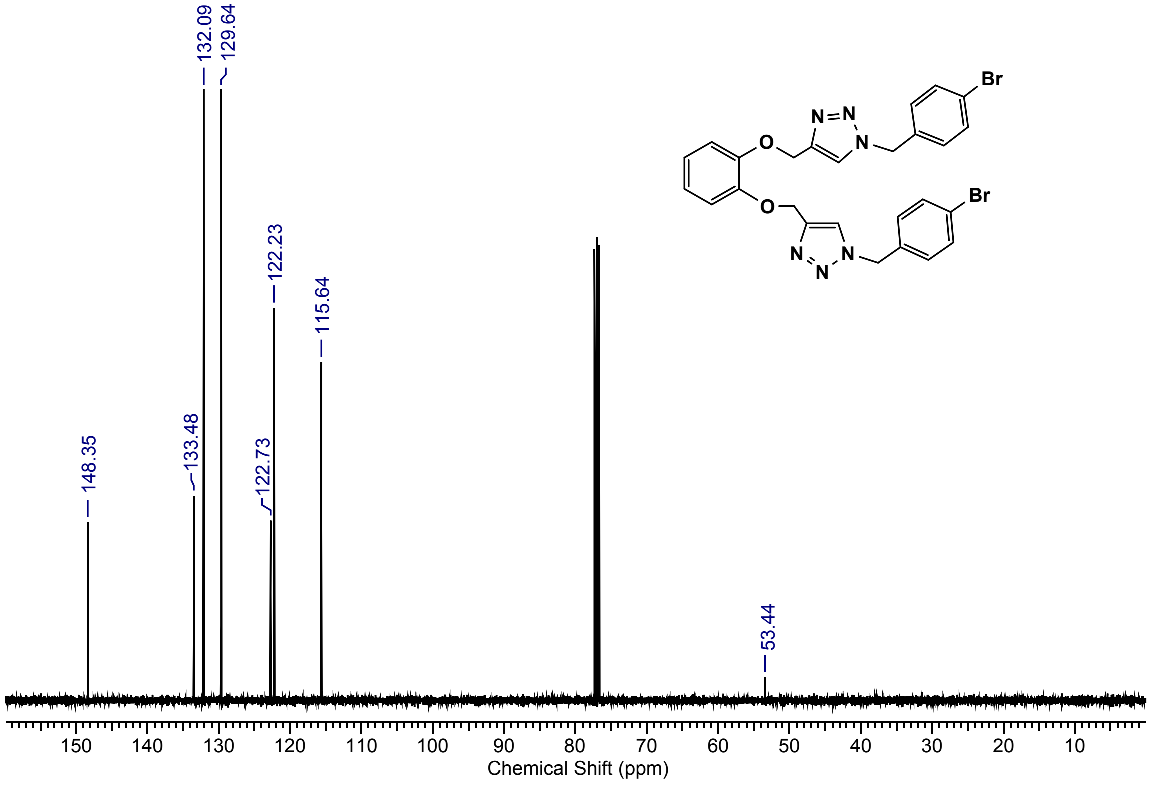

^{13}C-NMR spectrum of **25c**.

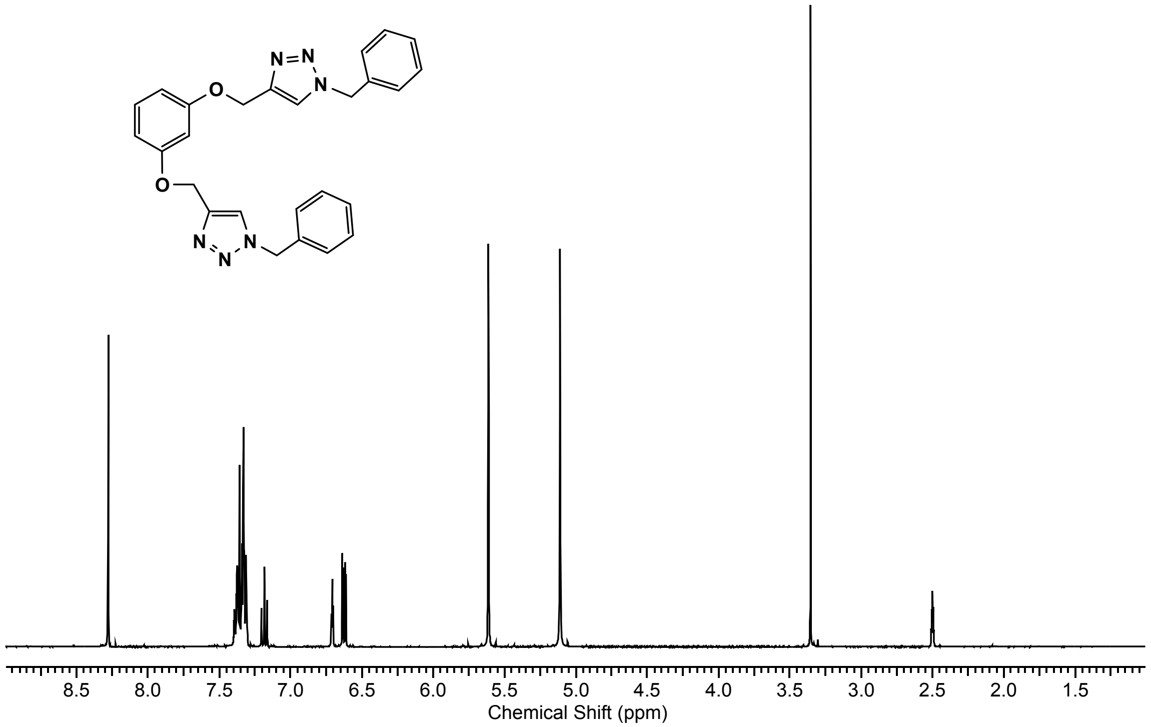

^{1}H-NMR spectrum of **triazole 26c**.

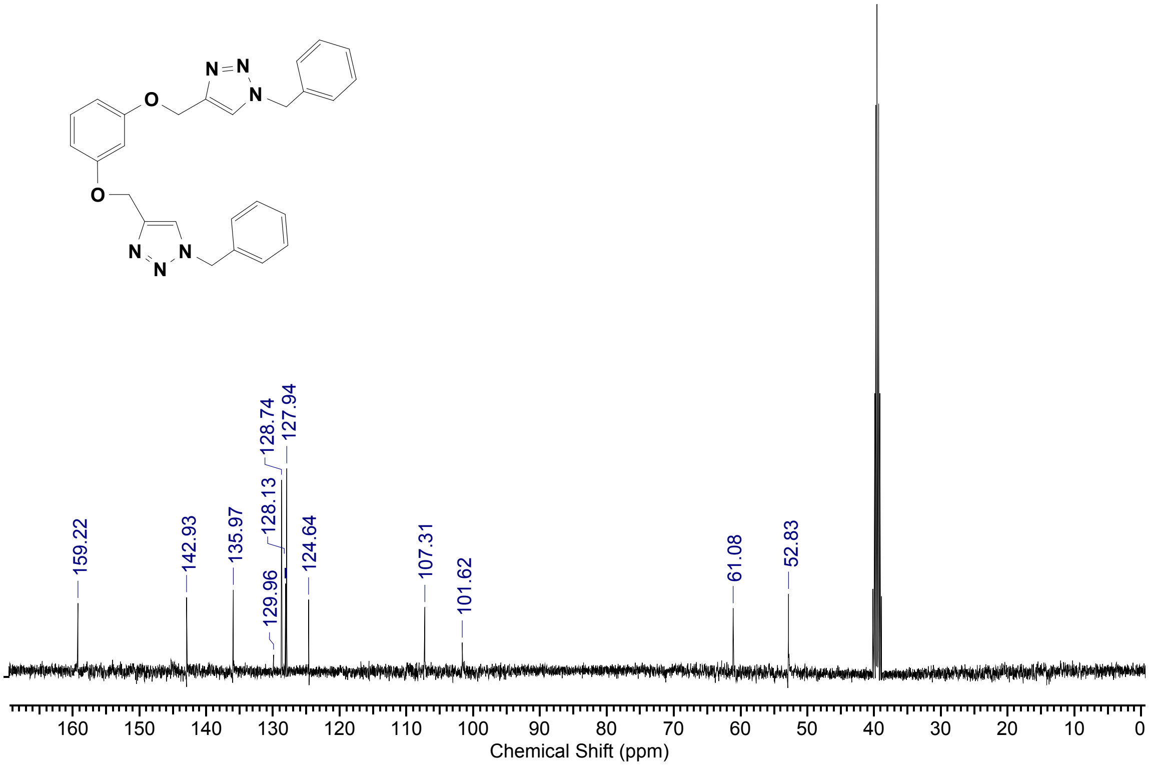

^{13}C-NMR spectrum of **triazole 26c**.

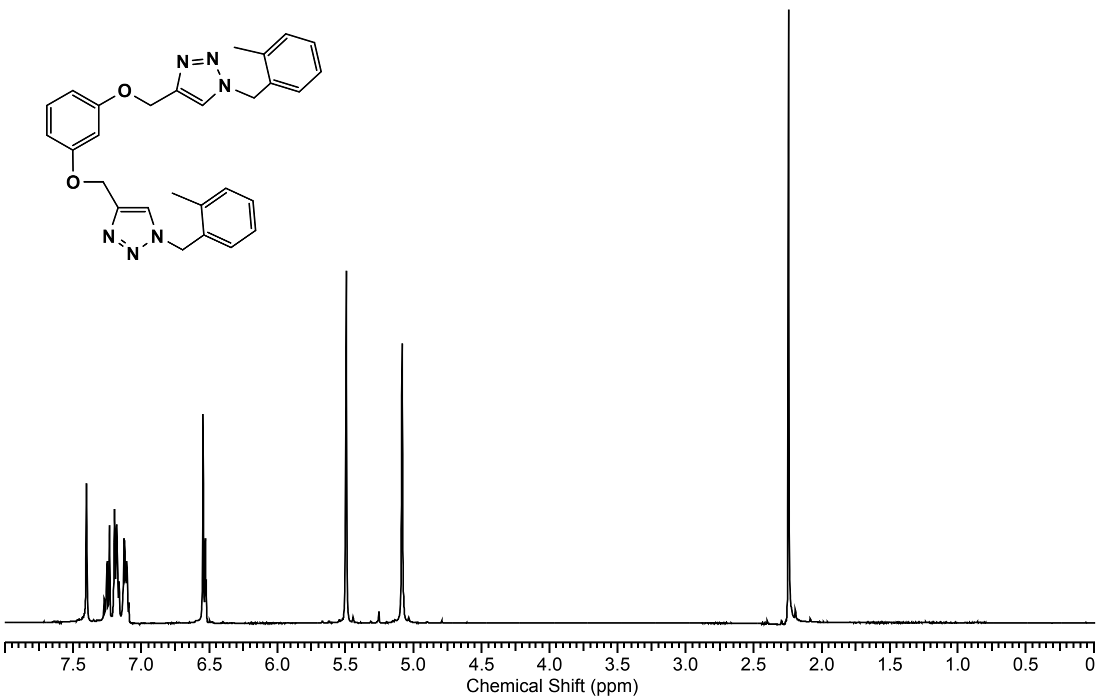

^{1}H-NMR spectrum of **27c**.

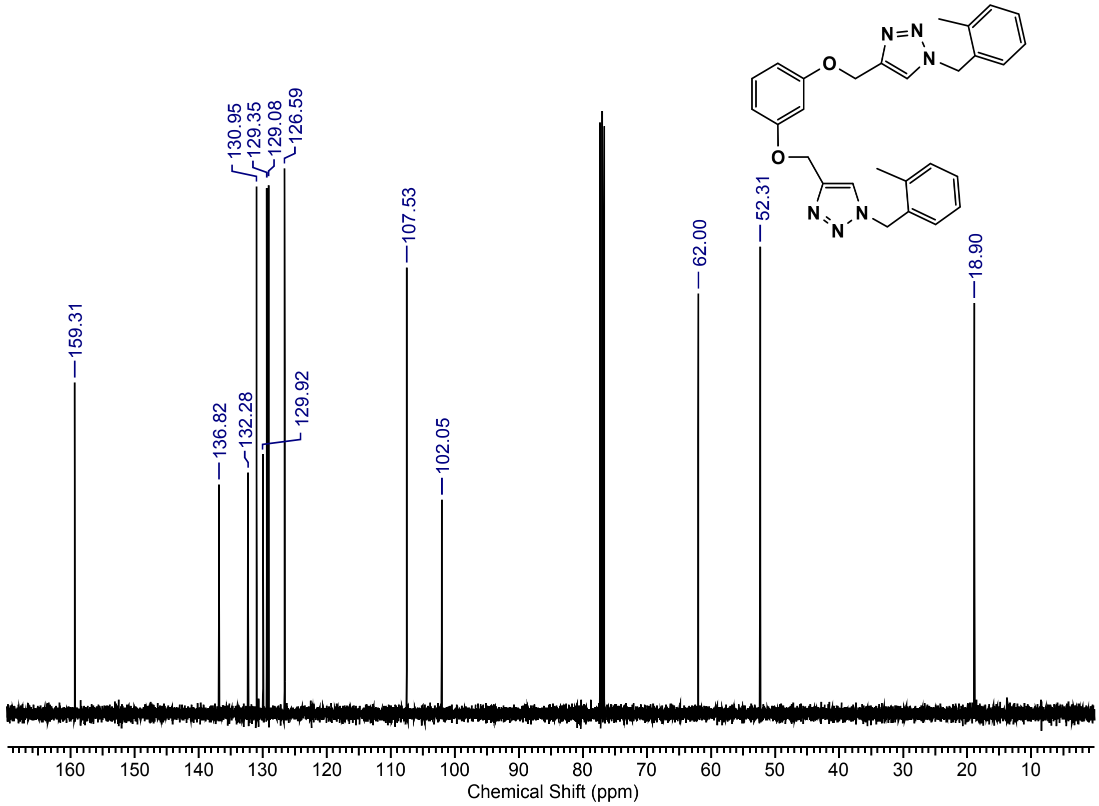

^{13}C-NMR spectrum of **27c**.

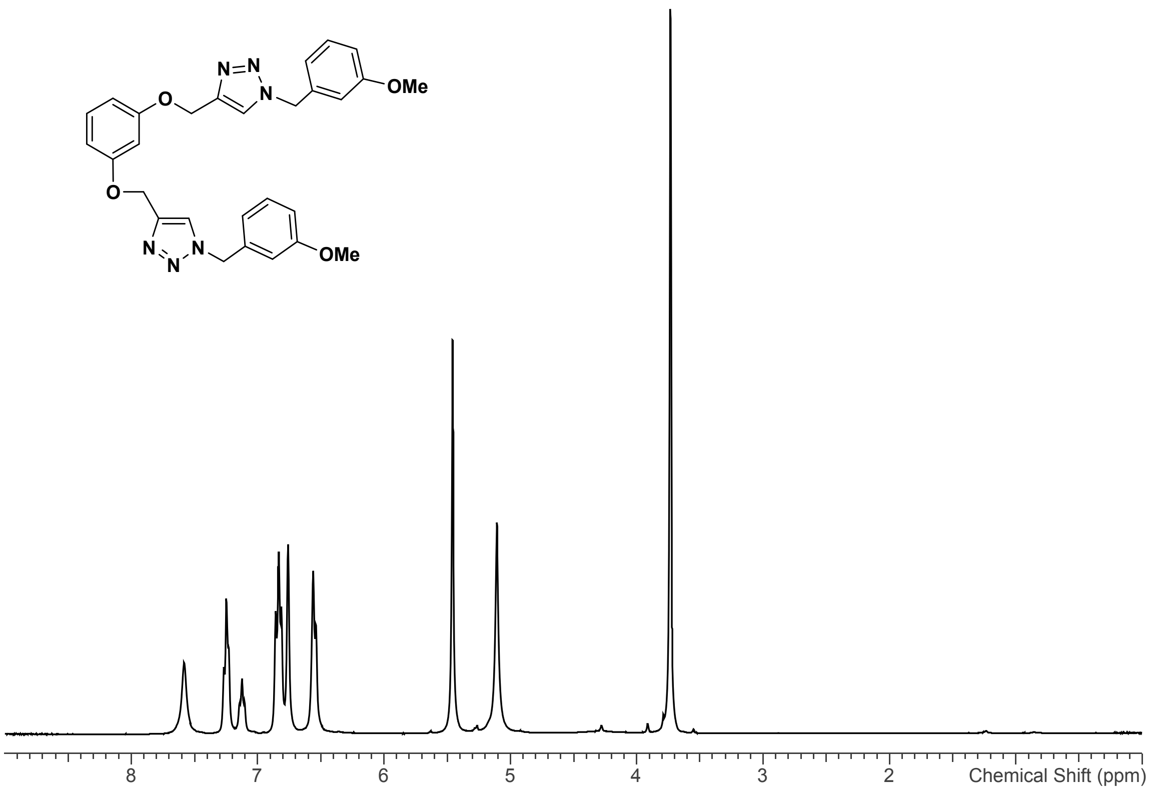

^{1}H-NMR spectrum of **28c**.

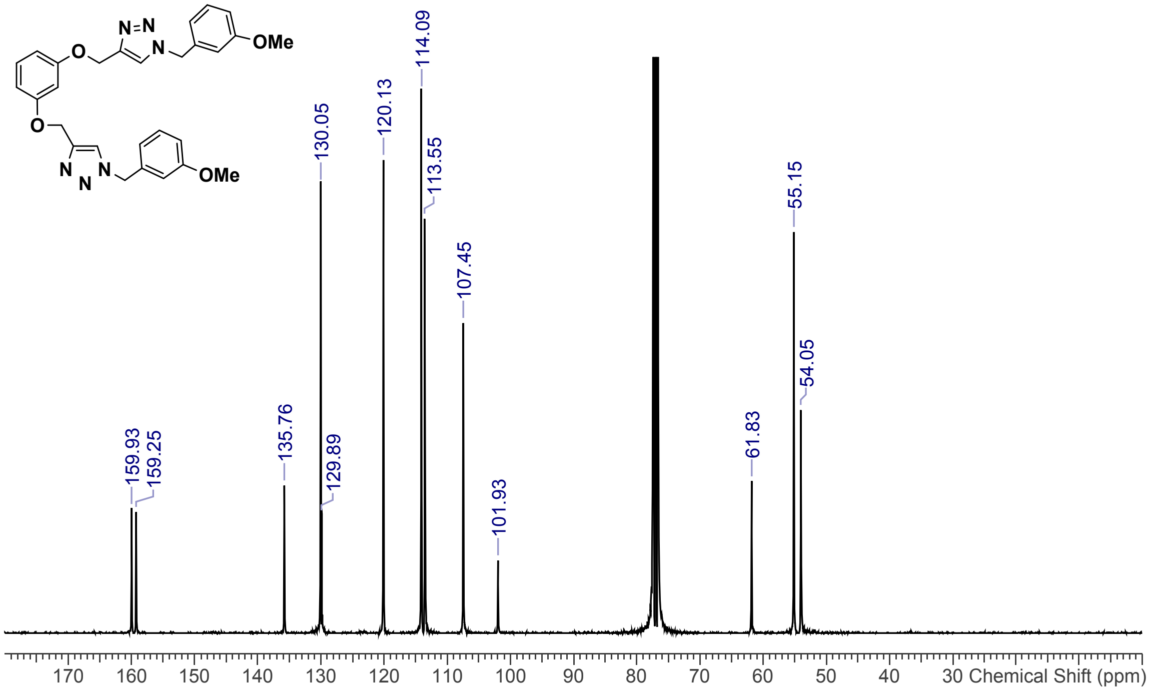

^{13}C-NMR spectrum of **28c**.

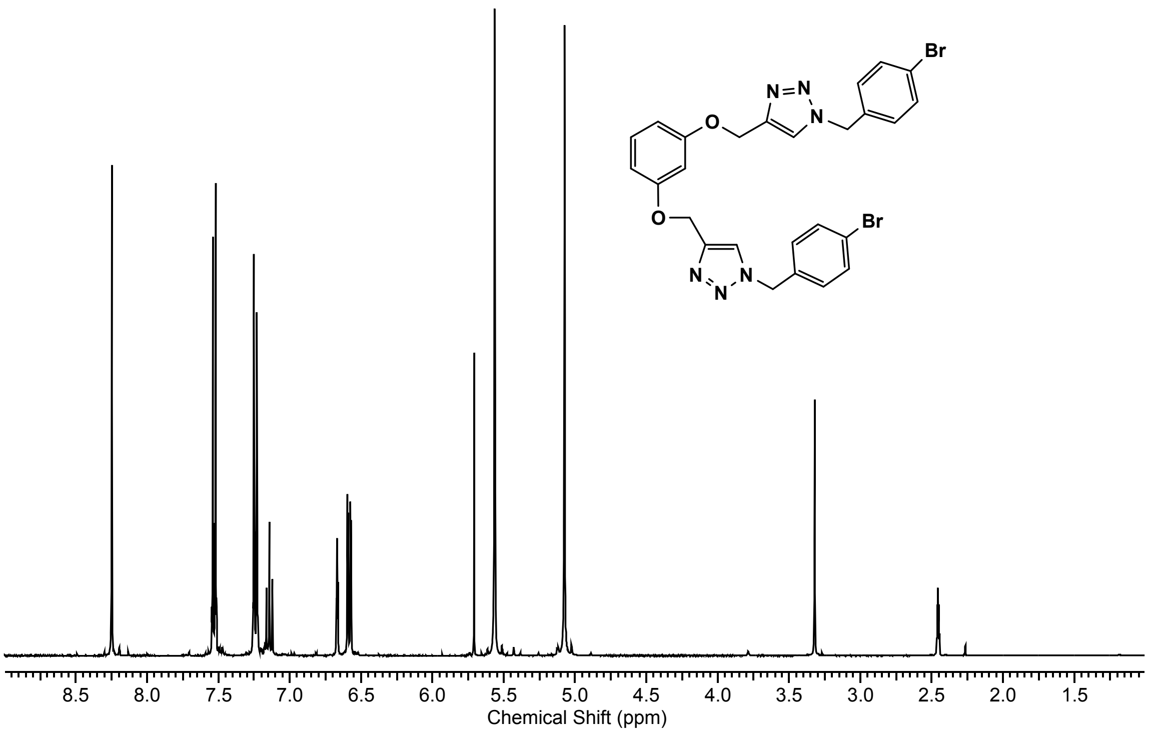

^{1}H-NMR spectrum of **29c**.

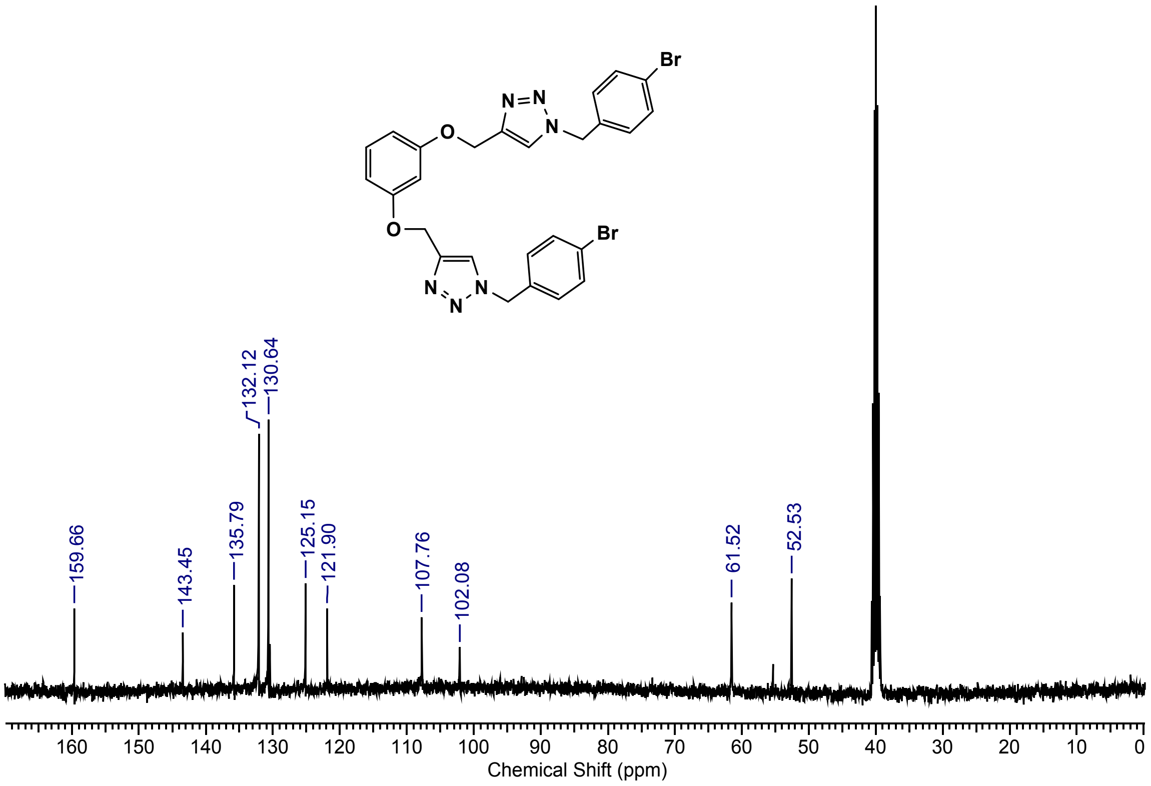

^{13}C-NMR spectrum of **29c**.

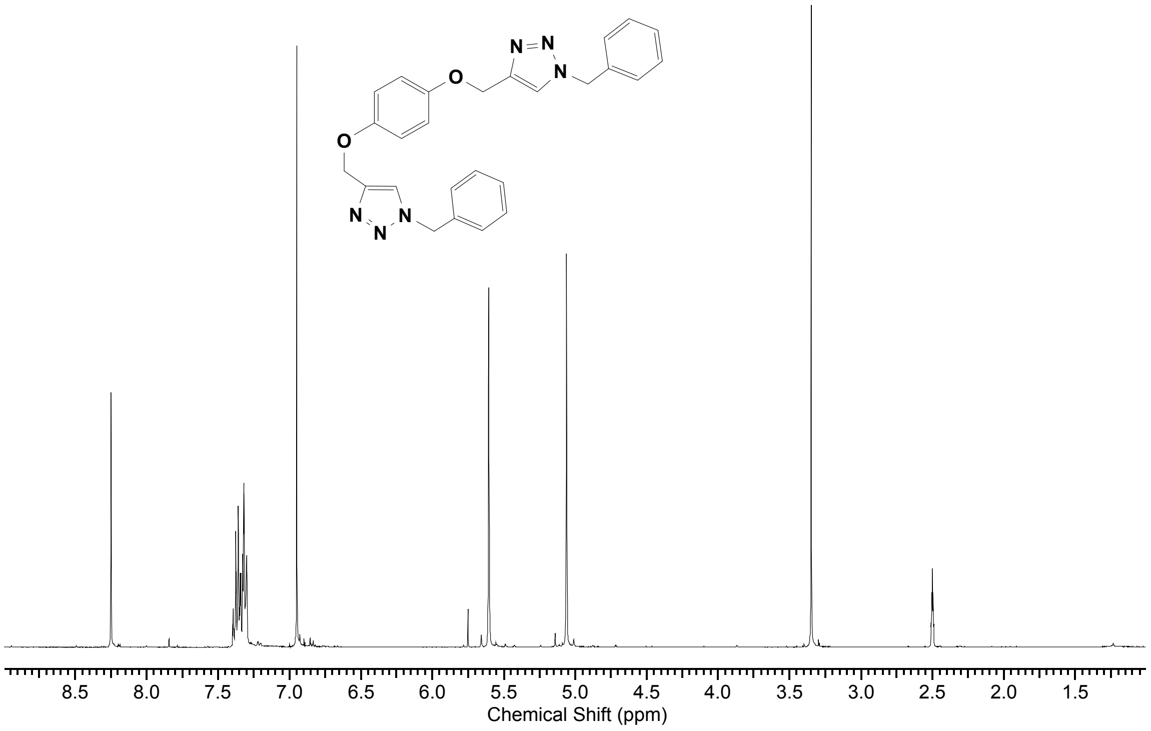

^{1}H-NMR spectrum of **30c**.

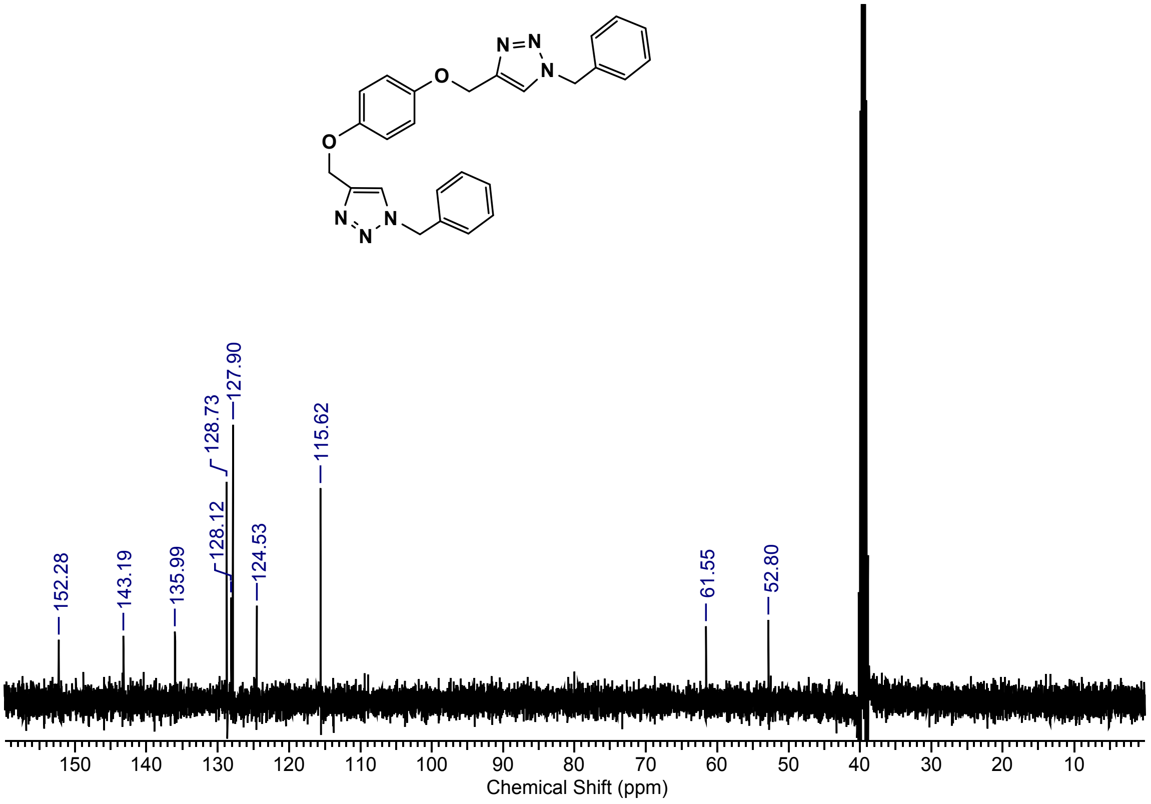

^{13}C-NMR spectrum, of **30c**.

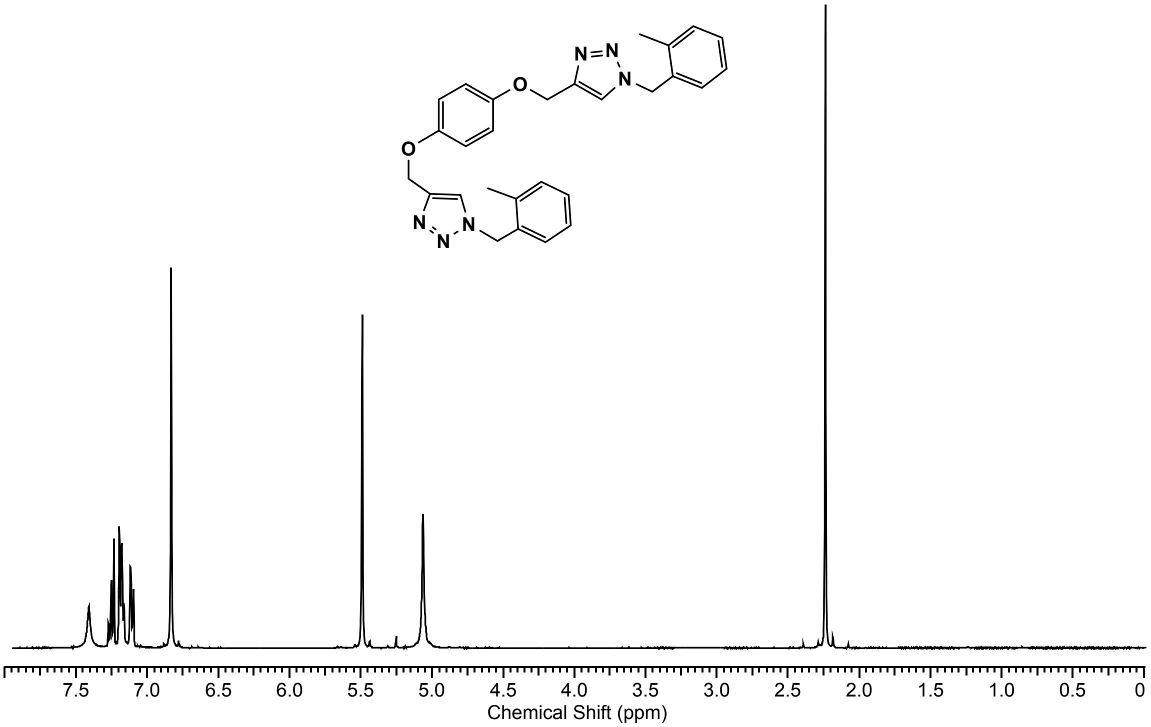

^{1}H-NMR spectrum of **31c**.

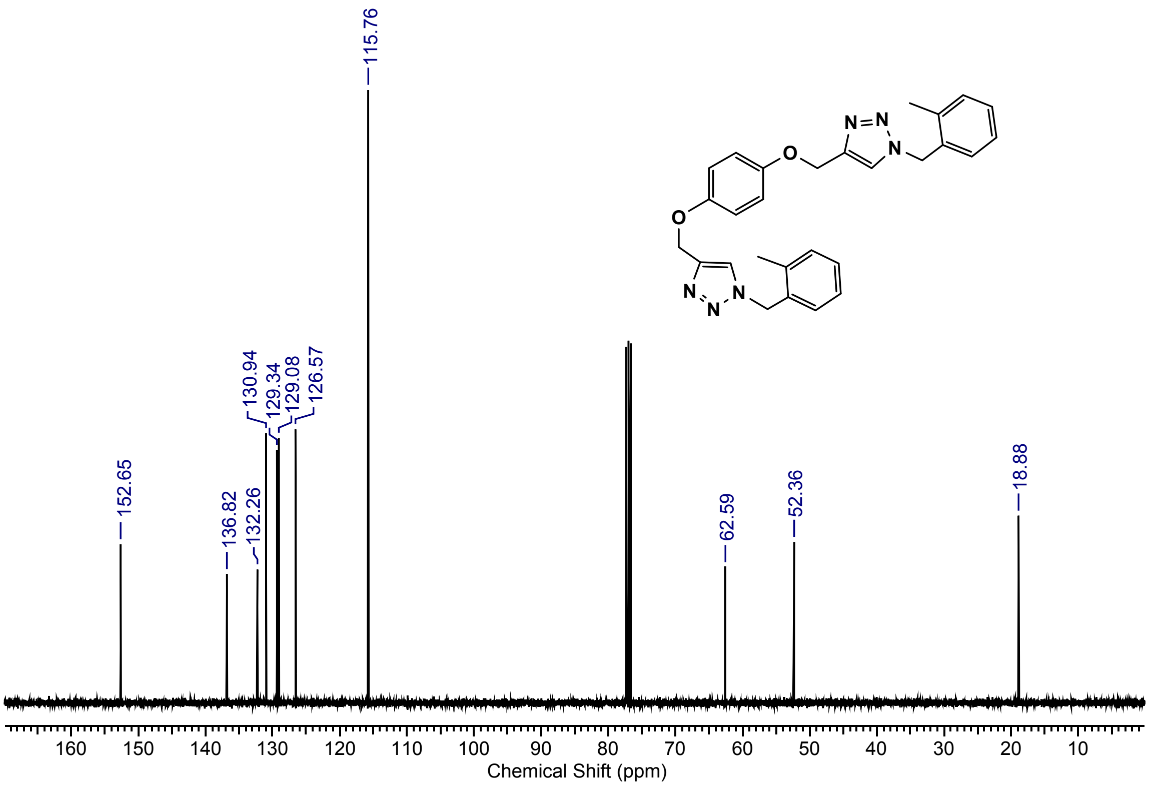

^{13}C-NMR spectrum of **31c**.

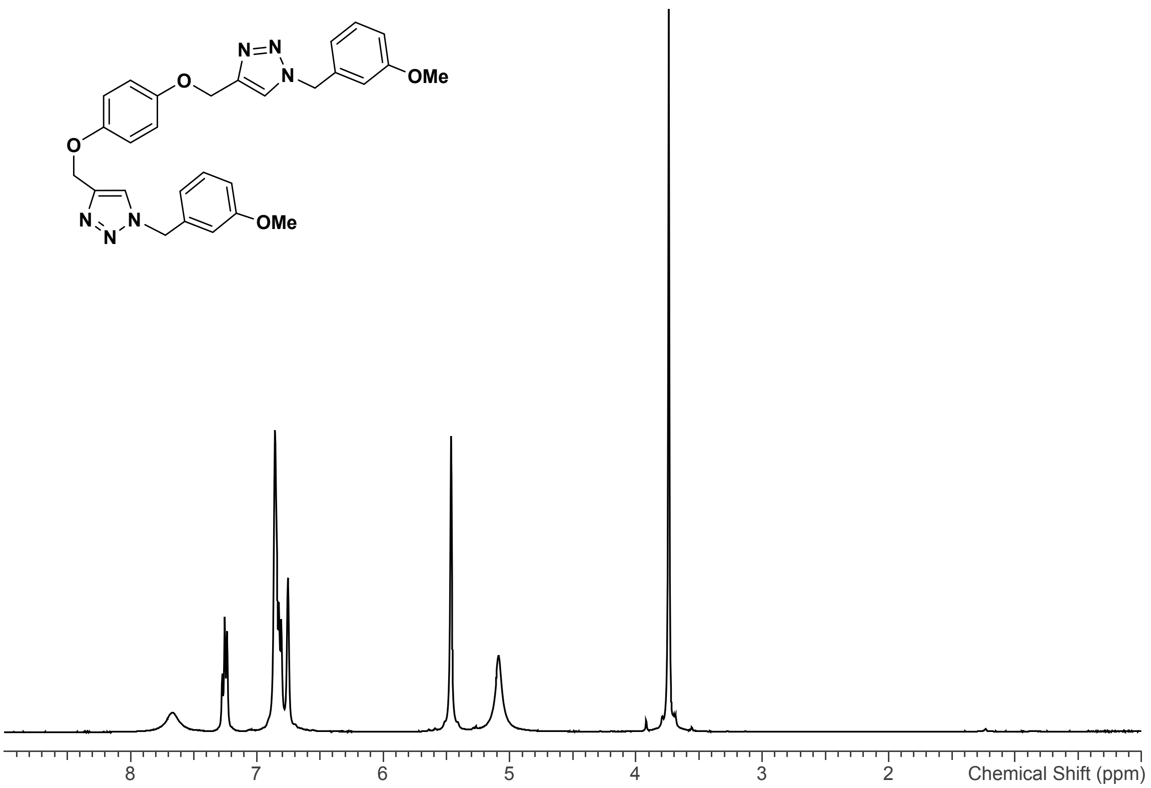

^{1}H-NMR spectrum of **32c**.

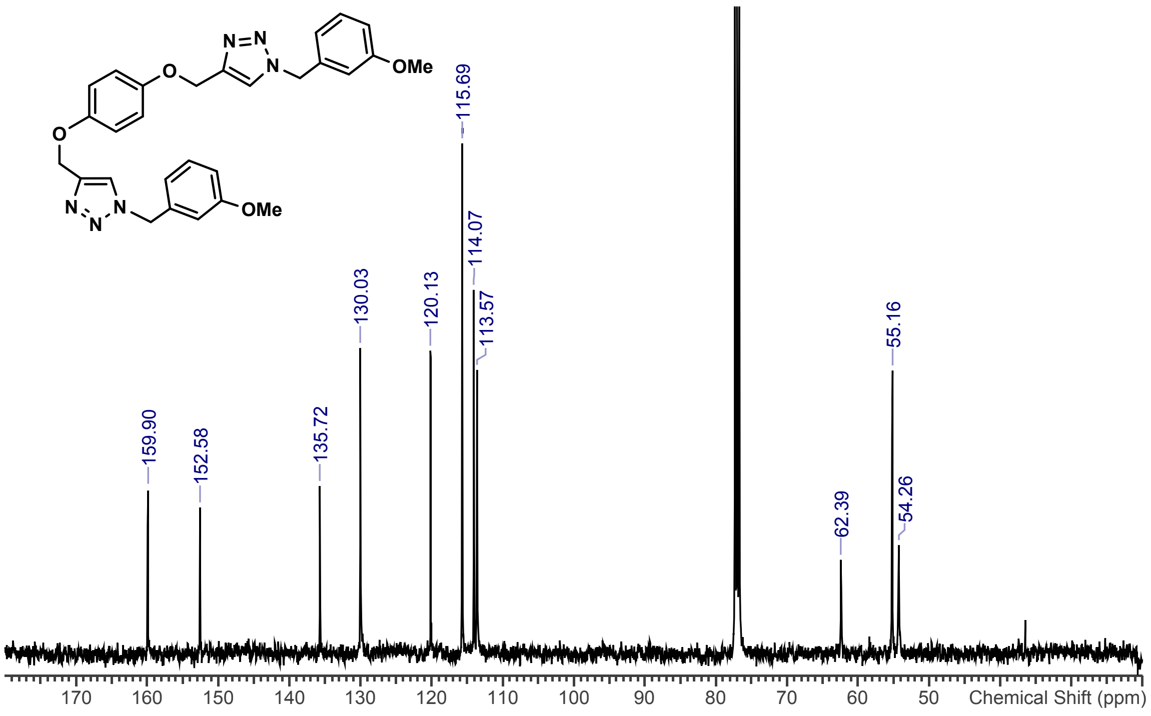

^{13}C-NMR spectrum of **32c**.

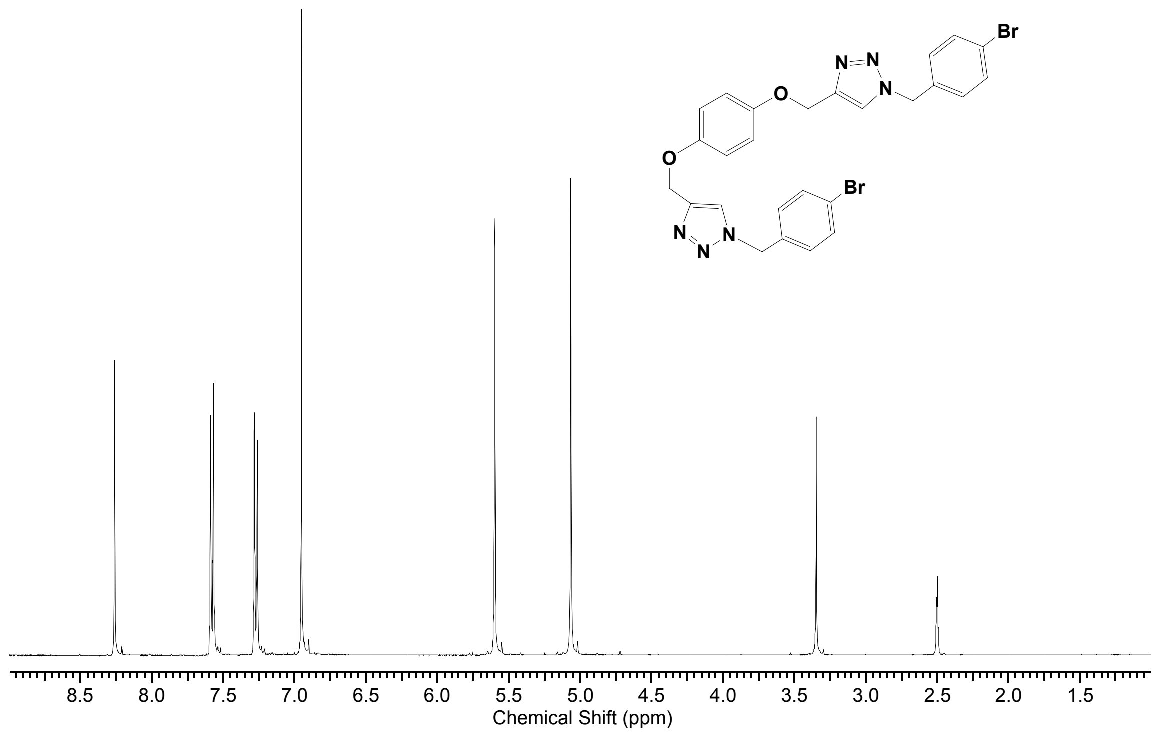

^{1}H-NMR spectrum of **33c**.

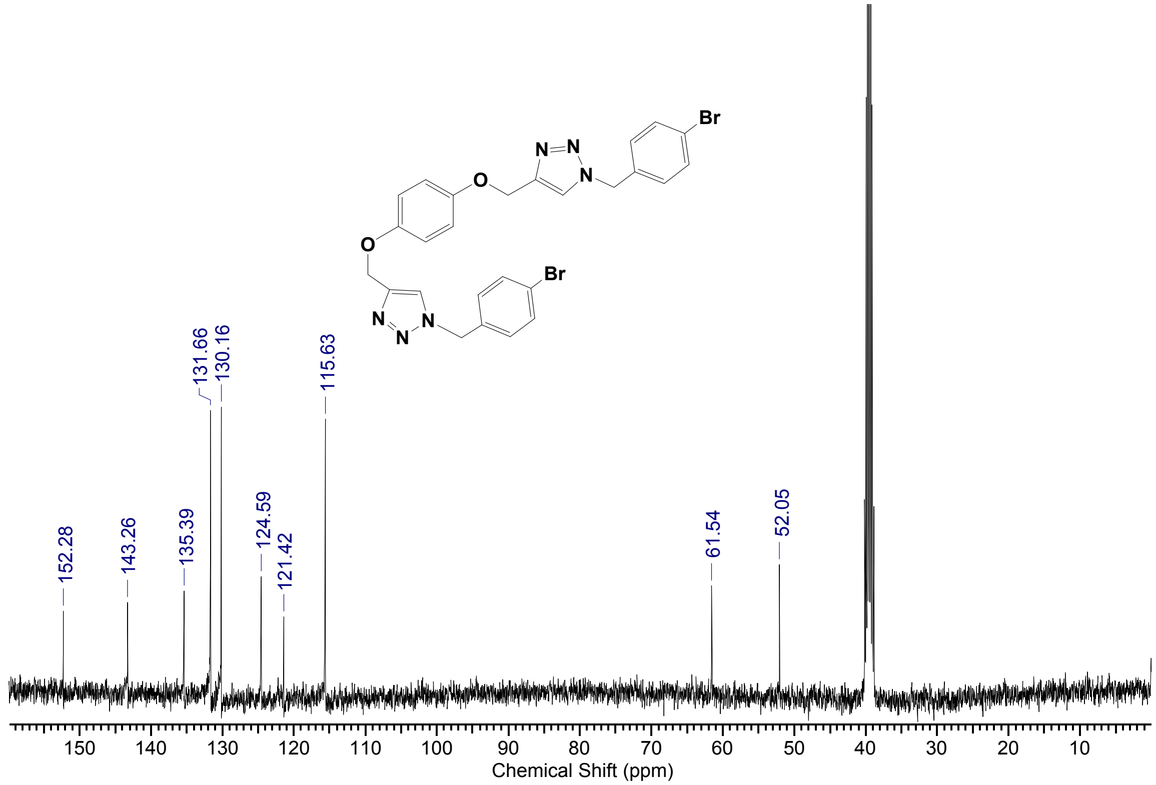

^{13}C-NMR spectrum of **33c**.

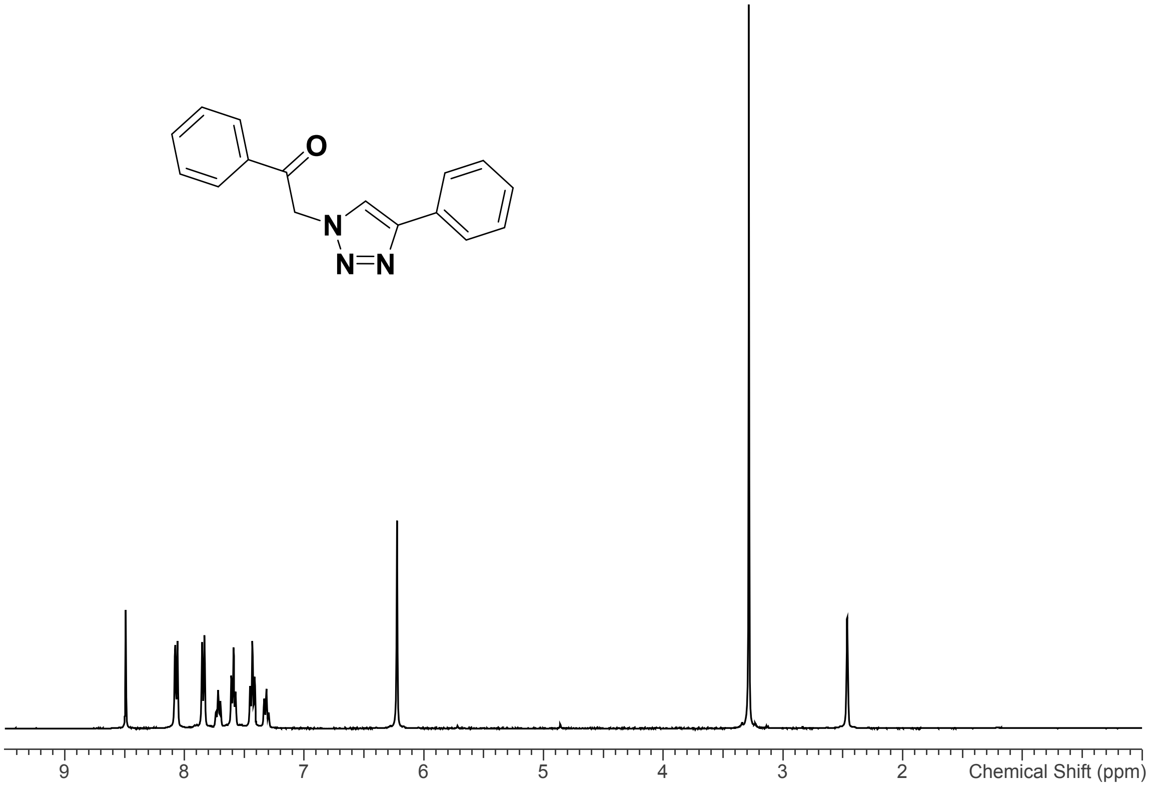

^{1}H-NMR spectrum of **triazole 35c**.

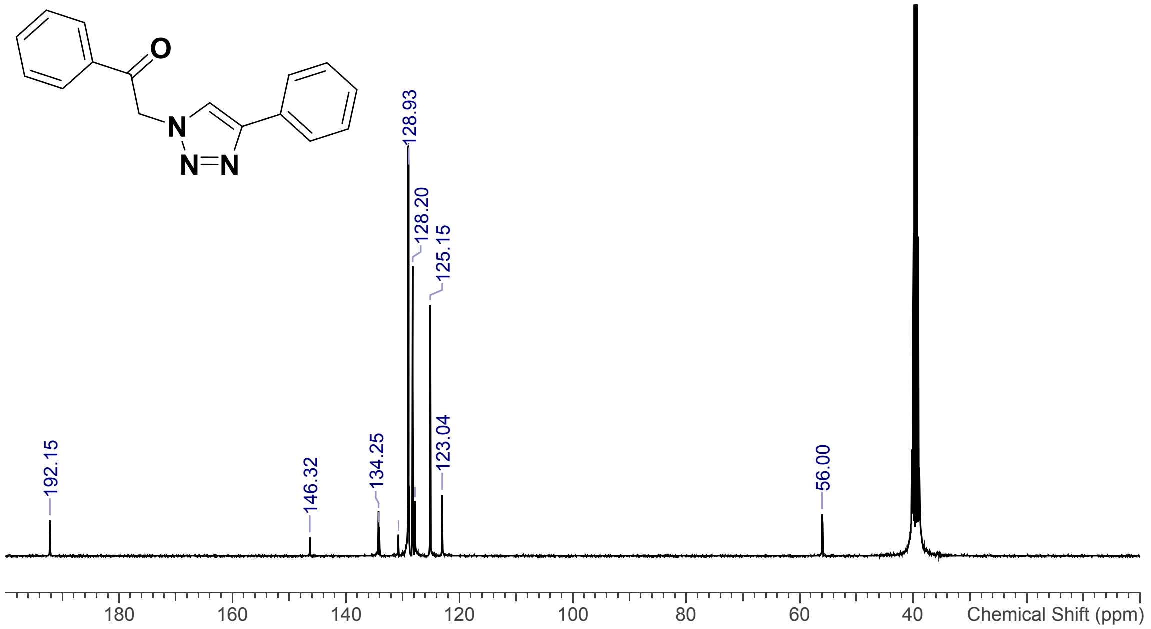

^{13}C-NMR spectrum of **triazole 35c**.

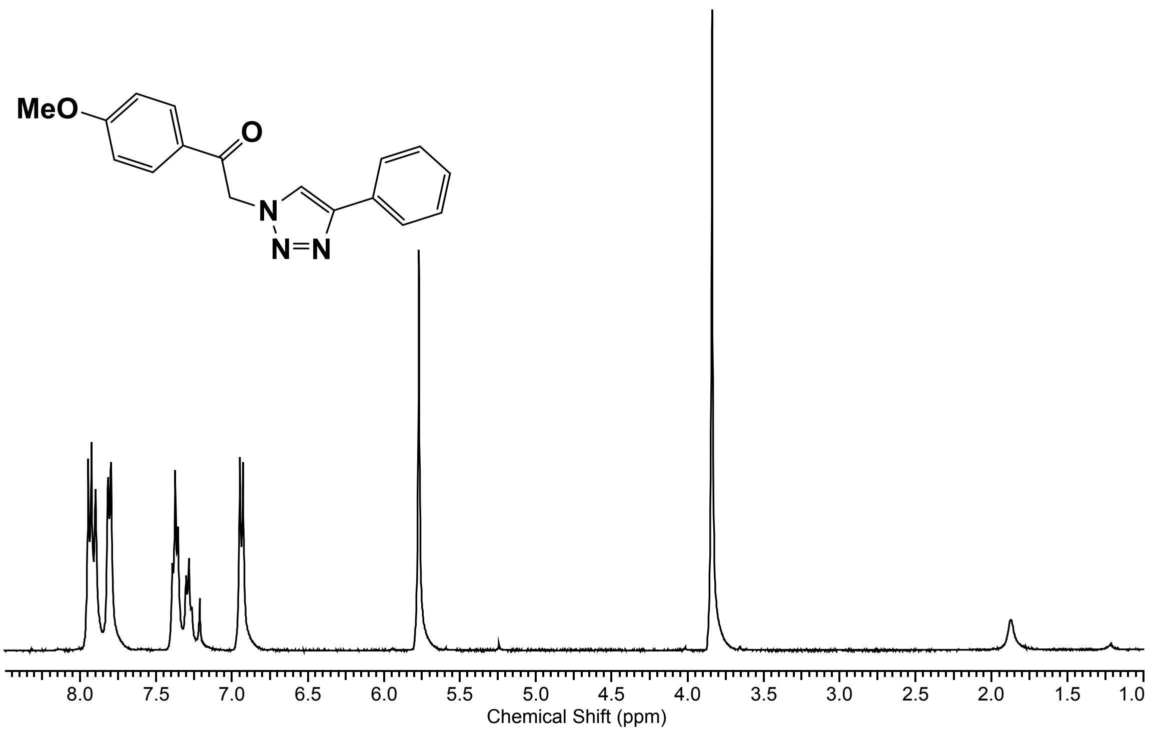

^{1}H-NMR spectrum of **triazole 36c**.

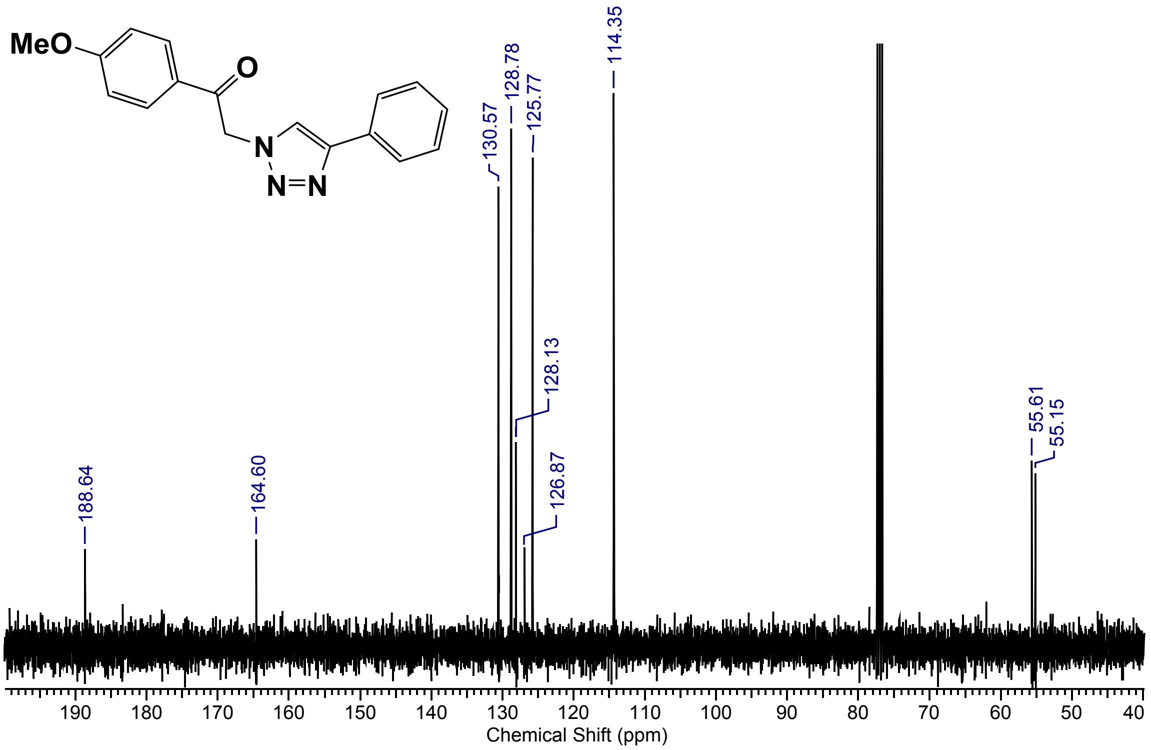

^{13}C-NMR spectrum of **triazole 36c**.
